# Aurora A kinase inhibition compromises its antitumor efficacy by elevating PD-L1 expression

**DOI:** 10.1172/JCI161929

**Published:** 2023-05-01

**Authors:** Xiaobo Wang, Jing Huang, Fenglin Liu, Qian Yu, Ruina Wang, Jiaqi Wang, Zewen Zhu, Juan Yu, Jun Hou, Joong Sup Shim, Wei Jiang, Zengxia Li, Yuanyuan Zhang, Yongjun Dang

**Affiliations:** 1 Key Laboratory of Metabolism and Molecular Medicine, the Ministry of Education, Department of Biochemistry and Molecular Biology, School of Basic Medical Sciences, Fudan University, Shanghai, China.; 2 Drug Discovery and Design Center, the Center for Chemical Biology, State Key Laboratory of Drug Research, Shanghai Institute of Materia Medica, Chinese Academy of Sciences, Shanghai, China.; 3 China University of Chinese Academy of Sciences, Beijing, China.; 4 Department of General Surgery and; 5 Department of Pathology, Zhongshan Hospital, Fudan University, Shanghai, China.; 6 Department of Pharmaceutical Sciences and Cancer Centre, Faculty of Health Science, University of Macau, Taipa, Macau SAR, China.; 7 Center for Novel Target and Therapeutic Intervention, Institute of Life Sciences, The Second Affiliated Hospital of Chongqing Medical University, Chongqing Medical University, Chongqing, China.

**Keywords:** Oncology, Cancer, Drug therapy

## Abstract

Aurora A plays a critical role in G_2_/M transition and mitosis, making it an attractive target for cancer treatment. Aurora A inhibitors showed remarkable antitumor effects in preclinical studies, but unsatisfactory outcomes in clinical trials have greatly limited their development. In this study, the Aurora A inhibitor alisertib upregulated programmed death ligand 1 (PD-L1) expression in a panel of tumor cells both in vitro and in vivo. Upregulation of the checkpoint protein PD-L1 reduced antitumor immunity in immune-competent mice, paradoxically inhibiting the antitumor effects of alisertib. Mechanistically, Aurora A directly bound to and phosphorylated cyclic GMP-AMP synthase (cGAS), suppressing PD-L1 expression in tumor cells. Aurora A inhibition by alisertib activated the cGAS/stimulator of IFN genes (STING)/NF-κB pathway and promoted PD-L1 expression. Combining alisertib with anti–PD-L1 antibody improved antitumor immunity and enhanced the antitumor effects of alisertib in immune-competent mice. Our results, which reveal the immunomodulatory functions of Aurora A inhibitors and provide a plausible explanation for the poor clinical outcomes with their use, offer a potential approach to improve the antitumor efficacy of these inhibitors.

## Introduction

Aurora A, a member of the evolutionarily conserved Aurora serine/threonine kinase family, is an essential mediator for centrosome maturation and separation and for the ultimate formation of the mitotic spindle during mitosis (1). Because of its central role in cell division, Aurora A amplification is found in a number of tumors and is known to be associated with poor clinical outcomes, making it an attractive target for cancer therapy (2). Inhibition of Aurora A induces growth inhibition and apoptosis in a variety of tumor cells (3). Several clinical trials of Aurora A inhibitors have been developed for cancer treatment (4). However, the only clinical trial to make it to phase III (alisertib, in patients with relapsed or refractory peripheral T cell lymphoma) was discontinued because of unsatisfactory efficacy (5). The reason that Aurora A inhibitors have failed to show benefits in patients with cancer is not yet clear. Recent studies have indicated a possible role for Aurora A in cancer-associated immunity (6, 7). However, the molecular details of how Aurora A is involved in the tumor microenvironment and in antitumor immunity are unknown. In addition, it is unclear whether the immune-regulatory function of Aurora A is involved in tumor resistance to Aurora A inhibitors.

Programmed death ligand 1 (PD-L1, also known as B7-H1 and CD274) is an immune checkpoint protein that is often overexpressed in a variety of human cancers (8). The interaction between PD-L1 and the programmed cell death 1 receptor (PD-1) suppresses the activity of tumor-infiltrating lymphocytes (TILs) and enables tumor cells to evade immune surveillance (9). Neutralizing antibodies targeting PD-1/PD-L1 have shown a substantial therapeutic benefit in patients with cancer (10–12). Anti–PD-1/anti–PD-L1 antibody (anti-PD) therapy has been integrated into the standard-of-care regimens for patients with multiple types of cancers, including advanced melanoma, renal cancer, hepatocellular carcinoma, and refractory Hodgkin lymphoma (13). Although anti-PD therapy has resulted in dramatic improvements in outcomes for some patients, many do not benefit from this therapy because of multifaceted primary and secondary resistance to immunotherapy (14, 15). In the clinic, PD-L1 levels are positively correlated with the immunotherapy response in patients with non–small cell lung cancer (NSCLC), colon cancer, and renal cell carcinoma (16–20). High PD-L1 expression levels are accompanied by an increase in TILs and are associated with a better response rate to anti-PD therapy (17).

In this study, we found that Aurora A kinase inhibitors increased PD-L1 expression in tumor and myeloid immune cells. In vivo, the Aurora A inhibitor alisertib compromised its own antitumor efficacy by upregulating PD-L1. Blocking PD-L1 with an anti–PD-L1 antibody enhanced the overall therapeutic efficacy of alisertib by augmenting T cell–mediated antitumor immunity. We also show that inhibition of Aurora A reduced cyclic GMP-AMP synthase (cGAS) phosphorylation. Unphosphorylated cGAS activated the stimulator of IFN genes (STING) pathway and its downstream transcription factor NF-κB, leading to the transcriptional upregulation of *PDL1*.

## Results

### Aurora A kinase inhibitors elevate PD-L1 expression in tumor cells.

We screened a compound library that included 69 kinase inhibitors using a high-throughput flow cytometry system based on PD-L1 expression (Supplemental Figure 1, A and B; supplemental material available online with this article; https://doi.org/10.1172/JCI161929DS1). Three aurora kinase inhibitors, the selective Aurora A inhibitor alisertib, the selective Aurora B inhibitor AZD1152, and the pan-Aurora inhibitor tozasertib, were found to enhance IFN-γ–induced PD-L1 expression (Supplemental Figure 1B and Supplemental Table 1). We validated these results with separate flow cytometric analyses for PD-L1 surface expression on BxPC3 cells stimulated with IFN-γ (Supplemental Figure 1, C and D). Treatment with aurora kinase inhibitors also enhanced IFN-γ–induced PD-L1 expression in other human tumor cell lines, including A549, HCT116, and T24 (Supplemental Figure 2, A and B).

**Figure 1:**
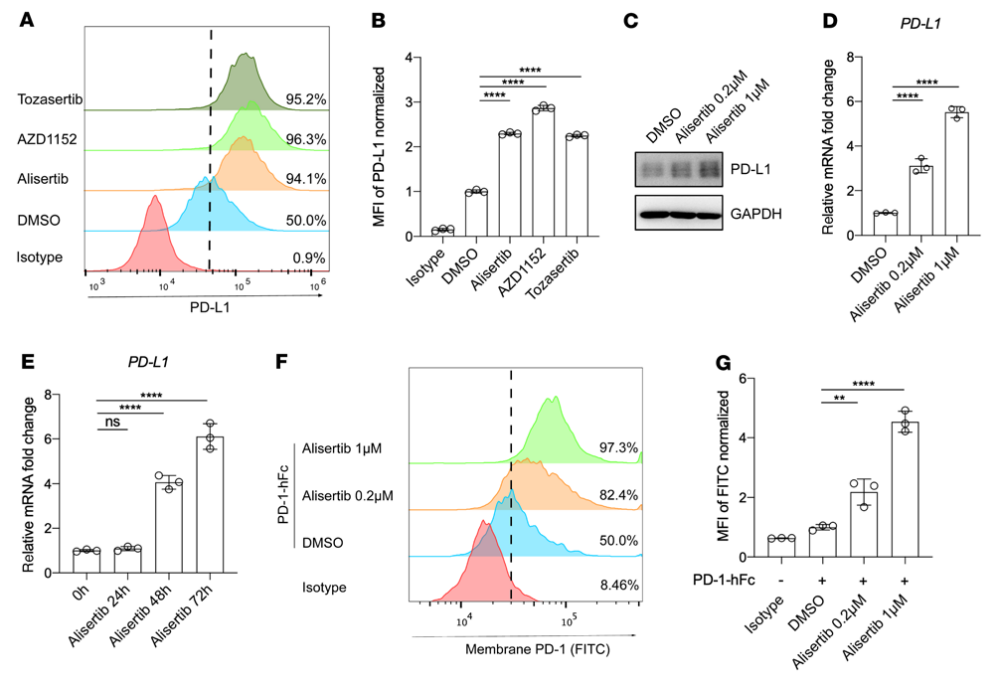
Aurora A kinase inhibitor elevates PD-L1 expression in tumor cells. (**A**) Flow cytometric analysis of cell-surface PD-L1 expression in BxPC3 cells following treatment for 72 hours with 1 μmol/L alisertib, 0.5 μmol/L AZD1152, or 0.5 μmol/L tozasertib. (**B**) Normalized MFI for the data shown in **A** (*n* = 3). (**C**) Western blot showing PD-L1 expression in BxPC3 cells after treatment for 72 hours with the indicated concentrations of alisertib. (**D** and **E**) qRT-PCR analysis of *PDL1* expression in BxPC3 cells after treatment with alisertib at the indicated concentrations (**D**) and for the indicated durations (**E**) (*n* = 3). (**F**) Flow cytometric analysis of cell-surface PD-1 binding of BxPC3 cells after treatment for 72 hours with the indicated concentrations of alisertib. (**G**) Normalized MFI for the data in **F** (*n* = 3). hFc, human Fc. Data indicate the mean ± SD. **P < 0.01 and ****P < 0.0001, by 1-way ANOVA.

To further investigate whether the aurora kinase inhibitors could influence constitutive PD-L1 expression levels, we treated the BxPC3, A549, HCT116, and T24 cells with alisertib, AZD1152, or tozasertib in the absence of IFN-γ. We found that the 3 inhibitors increased the expression of PD-L1 in the 4 cancer cells lines (Figure 1, A and B, and Supplemental Figure 2, C and D). These results indicated that Aurora kinase inhibitors could elevate PD-L1 expression in a wide range of tumor cell types, regardless of the presence or absence of IFN-γ. Given that alisertib had advanced further in clinical trials than the other 2 compounds (5), we focused on alisertib for our study.

Western blot analysis showed that PD-L1 protein levels were increased in BxPC3 cells in a dose-dependent manner with alisertib treatment (Figure 1C). *PDL1* mRNA levels also increased in a dose- and time-dependent manner with alisertib treatment (Figure 1, D and E). Furthermore, alisertib treatment increased the surface binding of PD-1 protein in BxPC3 cells (Figure 1, F and G). These results demonstrated that alisertib increased PD-L1 expression and augmented the binding of PD-1 on the tumor cell surface.

### Knockdown of Aurora A kinase elevates PD-L1 expression in tumor cells.

**Figure 2:**
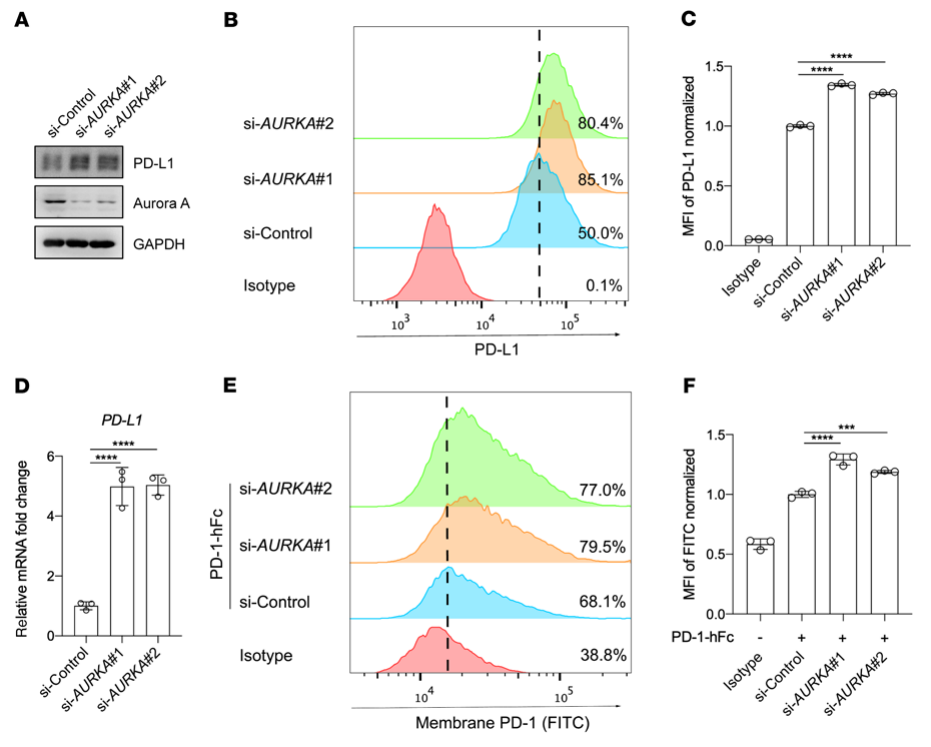
Knockdown of *AURKA* elevates PD-L1 expression in tumor cells. (**A**) Western blot analysis of the expression of indicated proteins following treatment with an *AURKA* siRNA or a control siRNA. (**B**) Flow cytometric analysis of surface expression of PD-L1 on BxPC3 cells after treatment with the indicated siRNA. (**C**) Normalized MFI for the data shown in **B** (*n* = 3). (**D**) qRT-PCR analysis of *PDL1* expression in BxPC3 cells after treatment with the indicated siRNA (*n* = 3). (**E**) Flow cytometric analysis of cell-surface PD-1 binding of BxPC3 cells after treatment with the indicated siRNAs. (**F**) Normalized MFI for the data in **E** (*n* = 3). Data indicate the mean ± SD. ***P < 0.001 and ****P < 0.0001, by 1-way ANOVA.

To determine whether PD-L1 upregulation was due specifically to the inhibition of Aurora A and was not an off-target effect of the inhibitor, we performed Aurora A kinase–knockdown (*AURKA*-knockdown)experiments in BxPC3 cells using 2 different siRNAs. Western blotting and flow cytometry confirmed that PD-L1 expression was substantially higher in *AURKA-*knockdown cells than in the scrambled control (Figure 2, A–C). In addition, *PDL1* mRNA levels and PD-1 binding also increased when *AURKA* was knocked down (Figure 2, D–F). These results demonstrated that the knockdown of *AURKA* also increased PD-L1 expression and augmented the binding of PD-1 on the tumor cell surface.

### Aurora A inhibition upregulates PD-L1 expression in vivo, which compromises the antitumor efficacy of the inhibitor.

**Figure 3:**
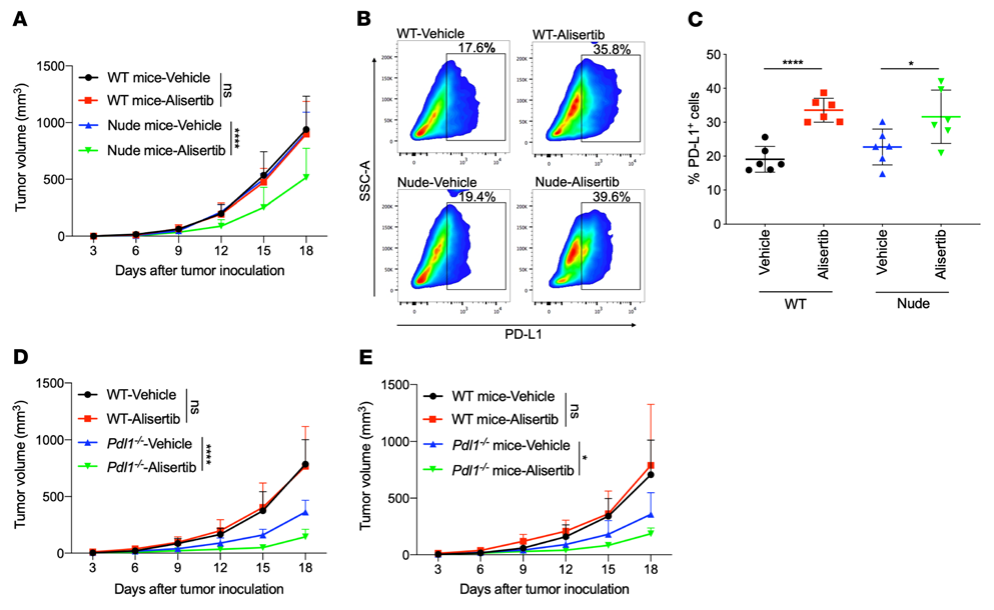
Aurora A inhibition upregulates PD-L1 expression in vivo, which compromises the inhibitor’s antitumor efficacy. (**A**–**C**) (**A**) Effect of vehicle or alisertib treatment on CT26 growth in BALB/c WT or BALB/c nude mice (*n* = 6). (**B**) Excised tumor tissues were digested to a single-cell suspension, and PD-L1^+^ cells were evaluated by flow cytometry. SSC-A, side scatter area. (**C**) Cumulative data for the percentage of PD-L1^+^ cells in **B** (*n* = 6). (**D**) Effect of vehicle or alisertib treatment on tumor growth in WT or *Pdl1^–/–^* MC38 mouse tumor models (*n* = 6). (**E**) Effect of vehicle or alisertib treatment on MC38 growth in WT or *Pdl1^–/–^* C57BL/6 mice (*n* = 6). Data indicate the mean ± SD. A 2-way ANOVA was applied to compare time-dependent tumor growth. *P < 0.05 and ****P < 0.0001, by unpaired *t* test.

We next determined whether Aurora A inhibition upregulates PD-L1 expression in vivo and affects tumor growth by regulating the tumor immune microenvironment. CT26 cells were implanted into immunocompetent BALB/c mice, as well as into immunodeficient BALB/c nude mice, and the antitumor effect of alisertib was assessed. Interestingly, pharmacological intervention with alisertib delayed tumor progression in immunodeficient BALB/c nude mice but failed to inhibit tumor growth in immunocompetent BALB/c mice (Figure 3A). Thus, the antitumor efficacy of alisertib was compromised in immune-competent mice.

Next, resected tumor tissues from both mouse types were dissociated, and PD-L1-expressing cells were measured by flow cytometry. As shown in Figure 3, B and C, the percentage of PD-L1^+^ cells increased substantially after the administration of alisertib in both BALB/c mice (vehicle/alisertib = 19.05%/33.55%) and BALB/c nude mice (vehicle/alisertib = 22.68%/31.62%). This is consistent with the observed increase in PD-L1 in 3 murine cell lines (CT26, MC38, and B16-F10) (Supplemental Figure 3, A–C). These results indicated that alisertib upregulated PD-L1 expression in vivo.

To test whether the alisertib-induced PD-L1 expression in tumor cells affected the function of cytotoxic T cells in the mouse tumor microenvironment, we performed immunohistochemical staining of cytotoxic T cells with granzyme B (21) in tumor tissues isolated from BALB/c mice. The results showed lower granzyme B staining intensity and a lower percentage of granzyme B^+^ cells in the alisertib-treated group than in the vehicle group (Supplemental Figure 4). These results indicated that alisertib reduced the activity of cytotoxic T cells.

To determine whether the compromised antitumor effect of alisertib was due to tumor PD-L1 induction in mouse tumor models, we generated 2 *Pdl1^–/–^* CT26 cell lines using the CRISPR/Cas9 system (Supplemental Figure 5A). Subsequently, WT CT26 cells or the 2 *Pdl1^–/–^* CT26 cells were implanted into immunocompetent BALB/c mice. The tumors in mice with implanted WT CT26 cells grew normally, whereas the tumors in mice implanted with *Pdl1^–/–^* CT26 cells showed spontaneous regression 6 days after implantation (Supplemental Figure 5, B and C). Thus, the *Pdl1^–/–^* CT26 cells were not suitable for evaluating the in vivo antitumor efficacy of alisertib. Next, we constructed a *Pdl1^–/–^* MC38 cell line (Supplemental Figure 6A) and implanted the cells into immunocompetent C57BL/6 mice. The results showed that tumors in *Pdl1^–/–^* MC38-implanted mice grew normally, although the tumor volume was smaller than that of tumors in WT MC38-implanted mice (Figure 3D and Supplemental Figure 6B). Then, we evaluated the antitumor effect of alisertib in the *Pdl1^–/–^* MC38 tumor models. Although alisertib failed to inhibit tumor growth in the WT MC38 group, it substantially delayed tumor progression in the *Pdl1^–/–^* MC38 group (Figure 3D and Supplemental Figure 6B). These results indicated that PD-L1 in tumor cells played an important role in restricting the in vivo antitumor effect of alisertib and that the compromised antitumor efficacy of alisertib was due to in vivo PD-L1 induction.

PD-L1 is expressed in many different host myeloid cells in addition to tumor cells, and the intensity of PD-L1 expression in certain types of host myeloid cells was higher than that of tumor cells. In addition, both host PD-1 and tumor PD-L1 contribute to immune suppression during the establishment of tumors (22–25). Therefore, we also assessed PD-L1 expression in macrophages (CD11b^+^F4/80^+^), myeloid-derived suppressor cells (MDSCs) (CD11b^+^Gr-1^+^), and DCs (CD11c^+^) with and without alisertib treatment (Supplemental Figure 7A). The results showed that PD-L1 expression increased in these immune cells after alisertib treatment, and the intensity of PD-L1 was much higher in macrophages, MDSCs, and DCs than in tumor cells (Supplemental Figure 7B). To further investigate whether PD-L1 expression on host myeloid cells contributes to reduced antitumor efficacy of alisertib, we evaluated the antitumor effect of alisertib in *Pdl1^–/–^* and WT mice. We observed no tumor inhibition in WT mice, but alisertib substantially inhibited tumor growth in the *Pdl1^–/–^* mice (Figure 3E and Supplemental Figure 6C).

Taken together, alisertib increased the expression of PD-L1 on both tumor cells and myeloid cells, and this increase strongly correlated with the impaired response to alisertib, as the loss of PD-L1 on tumor cells or systemically was associated with enhanced activity of Aurora A kinase inhibition.

### PD-L1 upregulation caused by Aurora A inhibition depends on STING/NF-κB activation.

**Figure 4:**
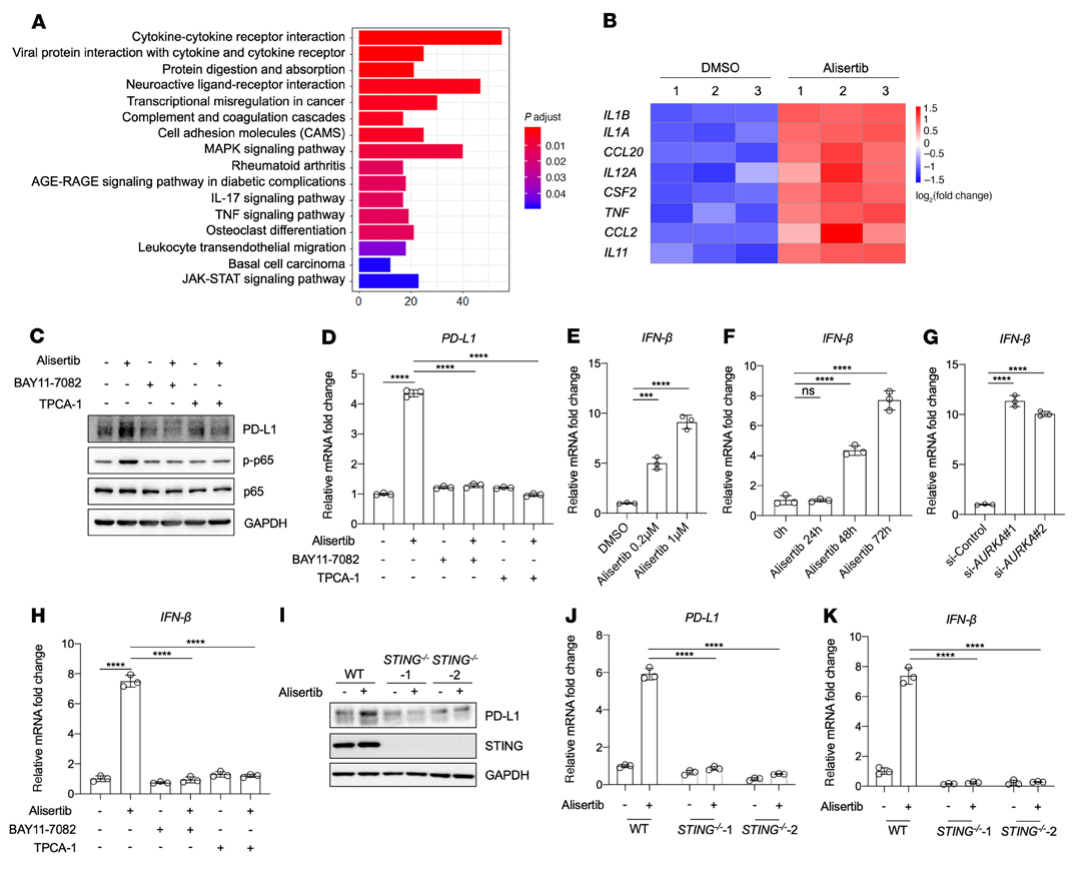
PD-L1 upregulation caused by Aurora A inhibition depends on STING/NF-κB activation. (**A** and **B**) RNA-Seq analysis was performed on BxPC3 cells after treatment for 72 hours with DMSO or 1 μmol/L alisertib. (**A**) KEGG pathway analysis of genes differentially expressed between the DMSO- and alisertib-treated groups. The most substantially enriched pathways are shown. p.adjust, adjusted *P* value. (**B**) Heatmap of gene expression levels of the indicated cytokines or chemokines in DMSO- or alisertib-treated BxPC3 cells. (**C** and **D**) BxPC3 cells were pretreated for 6 hours with 10 μmol/L TPCA-1 or with 5 μmol/L BAY11-7082, followed by treatment for 72 hours with 1 μmol/L alisertib, and PD-L1 expression was assessed by Western blotting (**C**) and qRT-PCR (**D**). (**E** and **F**) qRT-PCR analysis of *IFNB* expression in BxPC3 cells after the indicated concentrations (**E**) and durations (**F**) of alisertib treatment (*n* = 3). (**G**) qRT-PCR analysis of *IFNB* expression in BxPC3 cells after treatment with the indicated siRNA (*n* = 3). (**H**) qRT-PCR analysis of *IFNB* expression (*n* = 3). (**I**–**K**) WT BxPC3 cells or *STING^–/–^* BxPC3 cells were treated with 1 μmol/L alisertib for 72 hours. (**I**) Western blot analysis of PD-L1 and STING protein levels. qRT-PCR analysis of *PDL1* (**J**) and *IFNB* (**K**) mRNA levels (*n* = 3). Data indicate the mean ± SD. ***P < 0.001 and ****P < 0.0001, by 1-way ANOVA (**D**–**H**) and 2-way ANOVA (**J** and **K**).

To investigate the mechanism by which alisertib upregulates PD-L1 expression, we performed RNA-Seq and Kyoto Encyclopedia of Genes and Genomes (KEGG) enrichment analysis of genes differentially expressed between the alisertib-treated group and the DMSO-treated group. Affected pathways were predominantly associated with the cytokine–cytokine receptor interaction pathway (Figure 4A). Interestingly, we found that the gene expression signature of cytokines induced by alisertib treatment was highly similar to that of previously reported STING/NF-κB–regulated cytokines (Figure 4B) (26). The upregulation of these cytokines was validated by quantitative reverse transcription PCR (qRT-PCR) (Supplemental Figure 8).

To determine whether NF-κB is involved in alisertib-induced PD-L1 upregulation, we pretreated BxPC3 cells with and without BAY11-7082 or TPCA-1, two different NF-κB inhibitors (27, 28). The cells were then treated with alisertib, and the levels of PD-L1 and phosphorylated NF-κB p65 (p–NF-κB, referred to hereafter as p-p65) were analyzed. Alisertib increased the levels of PD-L1 and p-p65 in untreated cells, but these increases were completely abolished by BAY11-7082 and TPCA-1 treatments (Figure 4C). qRT-PCR analysis confirmed that increased *PDL1* mRNA expression was also blocked by the inhibition of NF-κB (Figure 4D). These results demonstrated that NF-κB activation was essential for the upregulation of PD-L1 caused by alisertib.

Next, we examined the role of STING in alisertib-induced PD-L1 upregulation. We first detected the expression of IFN-β, an indicator of STING pathway activation (29, 30), and observed that *IFNB* mRNA expression was increased in a dose- and time-dependent manner with alisertib treatment (Figure 4, E and F). Likewise, *IFNB* mRNA levels were also increased when *AURKA* was knocked down (Figure 4G). Moreover, the upregulation of *IFNB* mRNA induced by alisertib was abolished after pretreatment with BAY11-7082 or TPCA-1 (Figure 4H).

To further investigate the requirement for STING in the upregulation of PD-L1 induced by Aurora A inhibition, we knocked down *STING* and observed that PD-L1 upregulation by alisertib was almost completely abolished (Supplemental Figure 9, A and B). A similar inhibition of *IFNB* mRNA levels was also observed when *STING* was knocked down (Supplemental Figure 9C). We then generated 2 *STING^–/–^* BxPC3 cell lines and found that BxPC3 cells lacking STING did not exhibit alisertib-induced upregulation of PD-L1 and IFN-β (Figure 4, I–K, and Supplemental Figure 10). These results demonstrated that STING was necessary for alisertib-induced PD-L1 upregulation.

IFN-β is well known to induce the production of PD-L1 in tumor cells by activating the JAK/STAT signaling pathways (31). To investigate whether IFN-β also participates in alisertib-induced PD-L1 upregulation, we used an IFN-α/β receptor–neutralizing (IFNAR-neutralizing) antibody to block the function of IFN-β. As shown in Supplemental Figure 11A, the bioactivity of IFN-β was dose-dependently decreased by increasing the IFNAR–neutralizing antibody. Next, we pretreated BxPC3 cells with 5 μg/mL IFNAR-neutralizing antibody to completely abolish the function of IFN-β, followed by treatment with alisertib and detection of PD-L1 expression. The results showed that alisertib could still upregulate PD-L1 expression in a dose-dependent manner when the function of IFN-β was blocked, although the upregulation of PD-L1 by alisertib was partially inhibited (Supplemental Figure 11B). Besides, we also used baricitinib, a JAK-STAT inhibitor (32), which interrupted the signaling of IFN-β efficiently (Supplemental Figure 11C). Consistently, PD-L1 expression remained increased upon alisertib treatment even in the presence of baricitinib (Supplemental Figure 11D).

The STING pathway could also activate the phosphorylation of downstream TANK-binding kinase 1 (TBK1) and IFN regulatory factor 3 (IRF3) in addition to NF-κB (33, 34). We therefore examined whether the STING/TBK1/IRF3 pathway was activated upon alisertib treatment. No detectable phosphorylation changes in STING, TBK1, or IRF3 were observed. By contrast, these proteins were robustly phosphorylated by herring testis (HT) DNA, a well-known STING activator (35) (Supplemental Figure 12A). Next, we used the TBK1 inhibitor amlexanox (36) to test the involvement of the TBK1/IRF3 pathway in PD-L1 upregulation. We found that pretreatment with amlexanox failed to inhibit alisertib-induced PD-L1 and IFN-β upregulation (Supplemental Figure 12, B and C). We also analyzed RNA-Seq data for the mRNA expression levels of *CCL5*, *CXCX9*, *CXCL10*, and *CXCL11*, the target genes downstream of the STING/TBK1/IRF3 pathway (26), and found no elevation in their expression levels after alisertib treatment (Supplemental Figure 12D). The mRNA expression data were validated by qRT-PCR (Supplemental Figure 12E), which indicated that activation of the STING/TBK1/IRF3 pathway was not relevant to alisertib-induced PD-L1 upregulation, although STING itself was required.

The tumor suppressor p53 is phosphorylated by ataxia-telangiectasia mutated (ATM) upon DNA damage signaling and is delivered to STING to participate in the formation of an alternative STING signaling complex (26, 37). Aurora A phosphorylates p53, leading to Mdm2-dependent ubiquitination and degradation of p53 (38). Hence, it can be postulated that Aurora A inhibition could stabilize p53 and facilitates its complex formation with STING to promote downstream signaling. To test whether p53 participates in alisertib-induced, STING-mediated PD-L1 upregulation, we knocked down *P53* and applied alisertib treatment. However, no obvious differences were observed in PD-L1 or IFN-β expression levels (Supplemental Figure 13, A–C). We observed similar results in 2 *P53^–/–^* cell lines (Supplemental Figure 13, D–G). These results demonstrated that p53 was not involved in alisertib-induced PD-L1 upregulation.

### Aurora A inhibition–induced PD-L1 upregulation is mediated by cGAS dephosphorylation.

**Figure 5:**
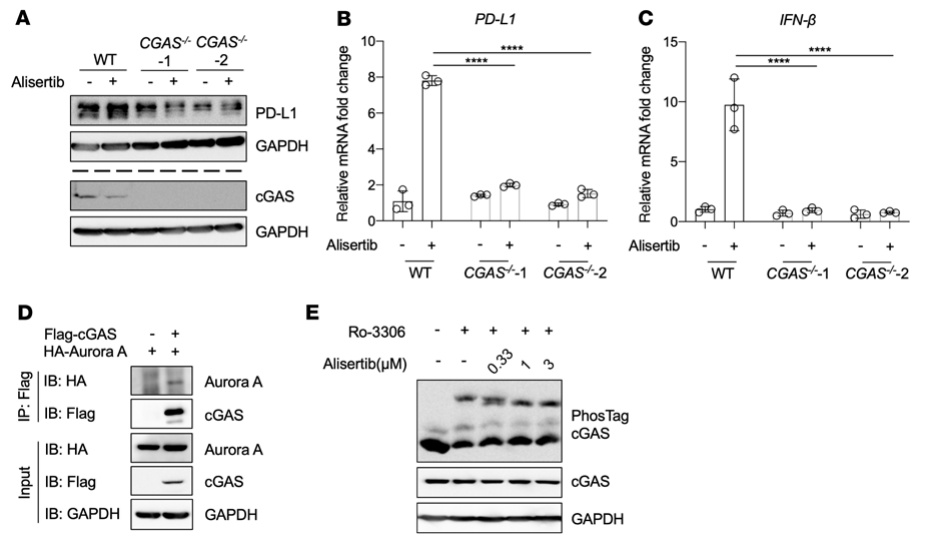
Aurora A inhibition–induced PD-L1 expression is mediated by cGAS dephosphorylation. (**A**–**C**) WT BxPC3 cells or *CGAS^–/–^* BxPC3 cells were treated with 1 μmol/L alisertib for 72 hours. (**A**) Western blot analysis of PD-L1 and cGAS protein levels. PD-L1 and cGAS were detected separately in 2 gels using the same biological samples, and GAPDH in each gel served as the loading control. qRT-PCR analysis of *PDL1* (**B**) and *IFNB* (**C**) mRNA levels (*n* = 3). (**D**) Co-immunoprecipitation of Aurora A and cGAS. HEK293T cells were transfected with the indicated vectors encoding HA–Aurora A and Flag-cGAS. Whole-cell lysates were immunoprecipitated with anti-Flag beads, and the interactions were analyzed by Western blotting. (**E**) BxPC3 cells were synchronized with 10 μmol/L Ro-3306 for 16 hours and released into mitosis in the presence of alisertib at the indicated concentrations. Phosphorylation of cGAS was analyzed by Phos-tag electrophoresis. Data indicate the mean ± SD. ****P < 0.0001, by 2-way ANOVA.

Given that PD-L1 upregulation induced by Aurora A inhibition depends on STING expression, we next explored a mediator that links Aurora A and STING. Because cGAS is a well-known upstream DNA sensor of STING and catalyzes the synthesis of 2′3′cyclic GMP-AMP (2′3′-cGAMP), a natural and efficient STING agonist (39), we first investigated the role of cGAS in alisertib-induced PD-L1 upregulation. We performed *CGAS* knockdown and evaluated PD-L1 expression by Western blotting and qRT-PCR after alisertib treatment. The results demonstrated that alisertib-induced PD-L1 upregulation was fully abolished (Supplemental Figure 14, A and B) and that the upregulation of IFN-β was also impaired (Supplemental Figure 14C). These results were confirmed using 2 *CGAS^–/–^* cell lines (Supplemental Figure 15). As shown in Figure 5, A–C, the upregulation of PD-L1 and IFN-β induced by alisertib was completely abolished in cells lacking cGAS. These results showed that cGAS was indispensable for alisertib-induced PD-L1 upregulation.

dsDNA was reported to activate cGAS in a sequence-independent manner (40, 41). To investigate whether alisertib-induced PD-L1 upregulation was due to dsDNA-induced cGAS activation, we detected the changes in cytosolic DNA levels after alisertib treatment but observed no obvious changes in cytosolic DNA concentration (Supplemental Figure 16). These results indicated that dsDNA was not involved in the alisertib-induced PD-L1 upregulation.

Aurora A is a kinase known to phosphorylate a number of cellular proteins, and cGAS is a cytosolic DNA sensor whose posttranslational modification, including phosphorylation, is critical for its regulation. We sought to explore the interaction between Aurora A and cGAS and the direct regulation of the activity of cGAS. Co-immunoprecipitation experiments with HA–Aurora A and Flag-cGAS showed that Aurora A bound to cGAS (Figure 5D). Next, to determine whether cGAS is a direct physiological substrate of Aurora A for phosphorylation, we performed Phos-tag electrophoresis, which detects phosphorylated proteins on the basis of their slower mobility through the gel (42). Since Aurora A expression and activity peak during the G_2_/M phase, we treated BxPC3 cells with different concentrations of alisertib after the cells were synchronized at the G_2_/M phase border by the CDK1 inhibitor Ro-3306. Ro-3306 treatment induced hyperphosphorylation of cGAS, which was determined by a high-molecular-weight mobility shift of the phosphorylated cGAS, indicating that cGAS was hyperphosphorylated during the G_2_/M phase when Aurora A was highly active (Figure 5E). The phosphorylation levels of cGAS were dose-dependently reduced by alisertib treatment (Figure 5E), suggesting that cGAS could be phosphorylated by Aurora A.

We next searched the cGAS protein sequence for a potential phosphorylation site that matched the Aurora A substrate consensus sequence: [KR]–[KR]–[S/Tp]–[Φ] (43), and a phosphorylation site was found at S64 in the cGAS N terminus. Next, we mutated the S64 residue to alanine (S>A) and aspartate (S>D) to mimic dephosphorylated and phosphorylated protein species, respectively, and induced overexpression of WT cGAS and mutant cGAS in *CGAS^–/–^* BxPC3 cells (Supplemental Figure 17A). The mRNA levels of *PDL1* and *IFNB* markedly increased when cGAS was aberrantly expressed. However, the upregulation of *PDL1* and *IFNB* mRNA expression levels was much lower in cells that overexpressed cGAS 64D than in cells that overexpressed cGAS 64A (Supplemental Figure 17, B and C). These results indicated that the activity of cGAS was inhibited when cGAS was phosphorylated at S64 by Aurora A, and that Aurora A inhibition resulted in dephosphorylated cGAS, which activated the cGAS/STING pathway to upregulate the expression of PD-L1 and IFN-β.

### Anti–PD-L1 therapy improves the antitumor efficacy of Aurora A inhibitors.

**Figure 6:**
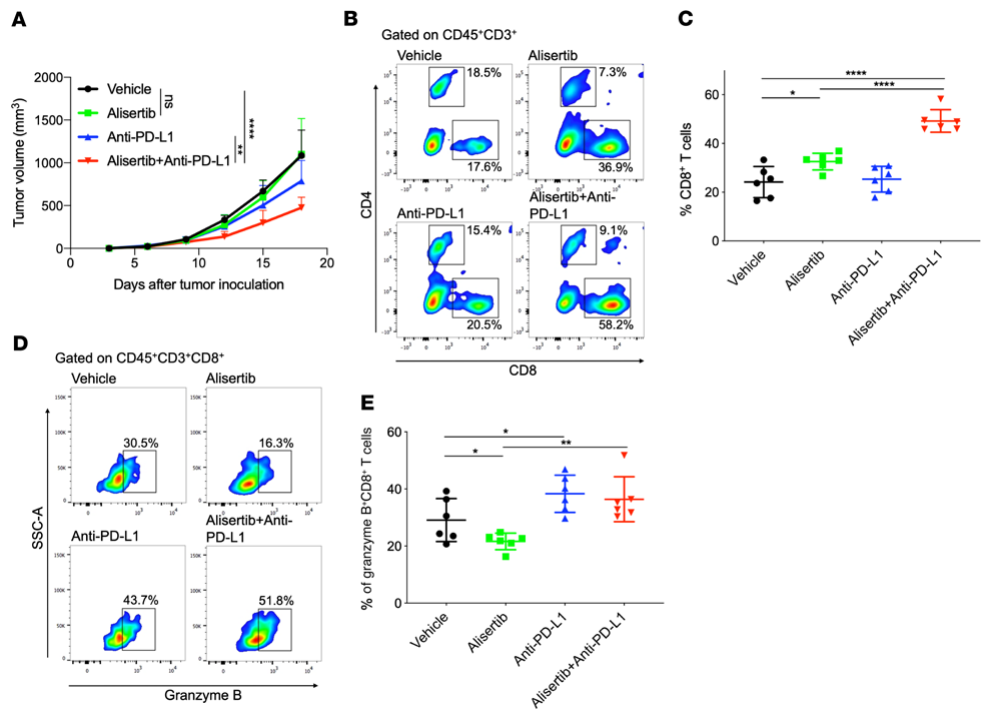
PD-L1 blockade therapy improves the antitumor activity of alisertib. (**A**–**E**) BALB/c mice were inoculated with CT26 cells and administered alisertib, anti–PD-L1 antibody alone, or their combination. (**A**) Tumor growth curves of CT26 in BALB/c mice. Tumor volumes were measured at the indicated time points (*n* = 6). (**B** and **D**) Flow cytometric analysis of tumor-infiltrating CD8^+^ T cells (**B**) and granzyme B^+^CD8^+^ T cells (**D**). Representative plots are shown. (**C** and **E**) Cumulative data for **B** and **D** (*n* = 6). Data indicate the mean ± SD. A 2-way ANOVA was applied to compare time-dependent tumor growth. *P < 0.05, **P < 0.01, and ****P < 0.0001, by unpaired *t* test.

To determine whether blocking the PD-L1 pathway could improve the antitumor efficacy of alisertib, BALB/c mice bearing CT26 tumors were administered alisertib and anti–mouse PD-L1 antibody alone or in combination. Mice treated with alisertib alone did not show a remarkable antitumor response, whereas treatment with an anti–PD-L1 antibody alone partially inhibited tumor growth. However, the combination of the 2 drugs substantially potentiated the antitumor effects of the anti–PD-L1 antibody in this model (Figure 6A and Supplemental Figure 18A). Furthermore, mice that received the combination treatment showed no marked changes in body weight (Supplemental Figure 18B), indicating that the mice were tolerant of the 2-drug therapy.

To evaluate antitumor immune responses in the local tumor microenvironment, we analyzed the changes in TILs in tumor tissues by multicolor flow cytometry. The infiltration of the total T cell population (CD3^+^) was markedly increased when the 2 drugs were combined (Supplemental Figure 18, C and D). Alisertib treatment alone increased the CD8^+^ T cell population compared with vehicle control treatment, and the alisertib-induced increase in the CD8^+^ T cell population was more pronounced when the inhibitor was combined with anti–PD-L1 antibody (Figure 6, B and C). The activity of tumor-infiltrating cytotoxic CD8^+^ T cells, as measured by granzyme B levels, was reduced after alisertib treatment, but the addition of anti–PD-L1 antibody restored this activity (Figure 6, D and E). In addition, alisertib treatment alone increased the infiltration of CD4^+^Foxp3^+^ Tregs, but the combined treatment with anti–PD-L1 antibody completely abolished the infiltration of CD4^+^Foxp3^+^ Tregs (Supplemental Figure 18, E and F). Next, we used anti-CD8 antibody to immunodeplete CD8^+^ T cells and observed that the antitumor effects of anti–PD-L1 antibody alone or in combination with alisertib were completely abolished (Supplemental Figure 19, A and B), which demonstrated the indispensable role of CD8^+^ T cells in the combination treatment. These results show that Aurora A inhibition by alisertib increased CD8^+^ T cell infiltration into tumors but decreased their cytotoxicity. Combination treatment with PD-L1 therapy overcame the adverse effect of alisertib on CD8^+^ T cells and potentiated the antitumor effects of alisertib.

### Active Aurora A levels are negatively associated with PD-L1 expression in human tumor tissues.

**Figure 7:**
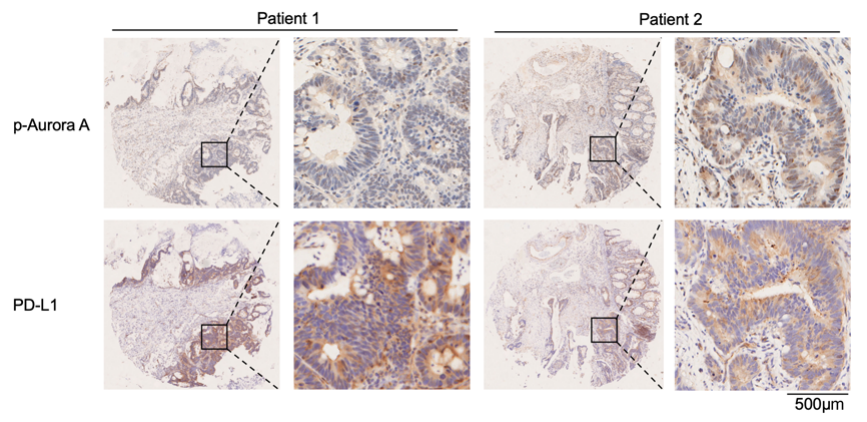
Active Aurora A levels are negatively associated with PD-L1 expression in human tumor tissues. Representative images of IHC staining for p–Aurora A and PD-L1 in human colon cancer tissues. Scale bar: 500 μm (enlarged insets).

We next explored the association of Aurora A activity and PD-L1 expression in samples from patients with cancer. Since Aurora A activity is dependent on its phosphorylation at the threonine 288 (T288) residue (44), we measured the levels of p–Aurora A at T288 and the expression levels of PD-L1 in human tissue microarrays. Tissue microarrays of 494 patients with colorectal carcinoma and corresponding para-carcinoma were examined using immunohistochemical staining. As shown in Supplemental Tables 2 and 3, p–Aurora A expression was detected in 139 carcinoma cases (28.1%) and 80 para-carcinoma cases (16.2%), and PD-L1 expression was detected in 63 carcinoma cases (12.8%) and 24 para-carcinoma cases (4.8%). We performed a correlation analysis of patient carcinoma tissues with positive p–Aurora A and PD-L1 expression. As a result, the expression levels of p–Aurora A were negatively associated with PD-L1 status (*P* = 0.04) (Supplemental Table 4). Specifically, approximately 63.2% of the carcinoma samples with low p–Aurora A expression had strong PD-L1 staining, and 83.3% of those with high p–Aurora A expression exhibited weak PD-L1 staining. Representative immunostaining images of p–Aurora A and PD-L1 are shown in Figure 7 and Supplemental Figure 20. These results indicated that the levels of active Aurora A were negatively associated with PD-L1 expression in human tumor tissues.

## Discussion

Currently, several Aurora A inhibitors are in clinical trials for cancer treatment. However, the most advanced clinical trial (phase III) of alisertib treatment for patients with relapsed or refractory peripheral T cell lymphoma has been discontinued due to unsatisfactory efficacy (5). Evaluations of the antitumor efficacy of Aurora A inhibitors have focused primarily on direct tumor killing, with little attention paid to cancer-associated immunity. Our study demonstrated that inhibition of Aurora A upregulated PD-L1 expression, thereby allowing tumor cells to escape from immune surveillance. This scenario describes a plausible mechanism that would explain the poor outcomes observed with the use of Aurora A inhibitors in clinical trials.

Our study unraveled the molecular mechanisms underlying the enhancement of PD-L1 expression by alisertib. Inhibition of Aurora A by alisertib caused cGAS dephosphorylation and activation, which subsequently activated the STING/NF-κB pathway and increased the transcriptional expression of *PDL1*. We also showed a direct interaction between Aurora A and cGAS and the phosphorylation of cGAS at S64 by Aurora A. The role of cGAS phosphorylation at S64 by Aurora A in the regulation of PD-L1 expression was explored by introducing phosphomimetic cGAS mutants (S64D), which showed a reduced ability to activate PD-L1 expression compared with nonphosphorylated mutants (S64A). A previous report showed that cGAS could be phosphorylated by Aurora B at S13 and S64 (45). The shared phosphorylation site on cGAS at S64 by the 2 aurora kinase isoforms indicated that Aurora B inhibitors might also upregulate PD-L1 expression by a similar mechanism. In this regard, the immune-suppressive effects of Aurora B inhibitors or pan–aurora kinase inhibitors might also be seen in vivo.

In the present study, we showed that the therapeutic efficacy of alisertib was compromised in MC38 mouse tumor models. However, alisertib inhibited the growth of *Pdl1^–/–^* MC38 cells in vivo and prevented the establishment of MC38 tumors in *Pdl1^–/–^* mice, which demonstrated that PD-L1 could compromise the in vivo therapeutic efficacy of alisertib. In addition, the increase in PD-L1 expression induced by alisertib impaired the cytotoxic function of T cells, although the infiltration of T cells was increased. Therefore, it is reasonable to use anti–PD-L1 antibody to reverse the adverse effects of alisertib mediated by PD-L1 induction. Yin et al. also demonstrated that alisertib treatment efficiently increases the number of infiltrated T lymphocytes, which cooperates with anti–PD-L1 therapy to inhibit the growth of 4T1 tumors (6). Our studies suggested a combination strategy to improve the antitumor efficacy of Aurora A inhibitors.

A recent report showed that nuclear Aurora A triggered the PD-L1–mediated immune suppression in triple-negative breast cancer (TNBC) cells (7). Nuclear Aurora A has recently emerged as an oncogene in certain types of tumors, and its function is largely distinct from that of the conventional Aurora A. Nuclear Aurora A has nonmitotic, kinase-independent functions and is involved in oncogene-mediated cell transformation and self-renewal of cancer stem cells (46, 47). Therefore, it can be postulated that tumors may have different regulatory mechanisms for immune surveillance depending on the presence of either nuclear or conventional Aurora A. Our study demonstrated an upregulation of PD-L1 expression not only by *AURKA* knockdown, but also by 3 different small-molecular Aurora kinase inhibitors in pancreatic, lung, melanoma, colorectal, and bladder cancer cells. This indicates that Aurora A kinase activity is involved in tumor immunity in a variety of tumor types.

In conclusion, we demonstrated that the selective Aurora A inhibitor alisertib upregulated PD-L1 expression, compromising its own antitumor efficacy. Further research revealed that upregulation of PD-L1 induced by alisertib occurred through activation of the cGAS/STING/NF-κB pathway. Our findings reveal the immunomodulatory functions of Aurora A, provide a plausible explanation for the poor clinical outcomes of Aurora A inhibitors in clinical trials, and suggest a combination strategy to overcome the adverse effects of Aurora A inhibitors.

## Methods

An expanded Methods section, including all uncut gels, is available in the supplemental materials.

### Mouse tumor model.

Six-week-old female BALB/c, BALB/c nude, and C57BL/6 mice were purchased from Shanghai SLAC Laboratory Animal Company. *Pdl1^–/–^* mice were purchased from the Shanghai Model Organisms Center. CT26/MC38 (5 × 10^5^ cells) or *Pdl1^–/–^* CT26/*Pdl1^–/–^* MC38 (1 × 10^6^ cells) cells were s.c. inoculated into the mice. For drug administration, alisertib (dissolved in 10% 2-hydroxypropyl-β-cyclodextrin and 1% sodium bicarbonate, oral gavage) was delivered once daily at 30 mg/kg. Anti–PD-L1 monoclonal antibody, dissolved in PBS (BE0101, clone 10F.9G2, Bio X Cell) was injected i.p. at 200 μg on days 6, 11, and 16. Anti-CD8 monoclonal antibody dissolved in PBS (BP0117, clone YTS169.4, Bio X Cell) was injected i.p. at 200 μg on days 1, 5, 9, 13, and 17. Mouse weights and tumor volumes were measured every 3 days. The tumor volume was calculated as follows: 1/2 × length × width^2^.

### Tumor-infiltrating cell analysis.

Tumors were excised at the endpoint, cut into small pieces, and digested with 1 mg/mL collagenase (MilliporeSigma) for 40 minutes in a 37°C shaking incubator. Cell suspensions were filtered through a 70 μm cell strainer, and RBCs were removed using Red Blood Cell Lysis Buffer (B541001, Sangon Biotech) according to the manufacturer’s protocol. Cells were collected, and PD-L1 expression and TILs were analyzed by flow cytometry.

### RNA-Seq and data analysis.

Total RNA isolated from the BxPC3 cell line was used to prepare cDNA libraries, which were subsequently sequenced on the Illumina HiSeq 2000 platform using the paired-end method. The sequencing reads were mapped to mm10 using STAR 2.5, and feature counts software was used to quantify gene expression. The EdgeR R package was used to perform differential gene expression analysis. The raw sequencing data reported in this work have been deposited in the Genome Sequence Archive (GSA) (48) at the National Genomics Data Center (49), China National Center for Bioinformation/Beijing Institute of Genomics, Chinese Academy of Sciences (GSA human accession number: HRA004057; https://ngdc.cncb.ac.cn/gsa-human/browse/HRA004057).

### Co-immunoprecipitation.

HEK293T cells were transfected with the indicated plasmids for 48 hours. Cells were washed in PBS and lysed in lysis buffer (20 mM Tris-HCl [pH 7.5], 150 mM NaCl, 1 mM EDTA, and 1% Triton X-100) supplemented with protease inhibitors (pepstatin, leupeptin, aprotinin, and PMSF) and phosphatase inhibitors (NaF and Na_3_VO_4_) for 30 minutes on ice. The lysates were centrifuged at 12,000 rpm (13,800*g*) for 15 minutes at 4°C to remove debris. The supernatants were incubated with anti-Flag sepharose beads at 4°C. After 3 hours of incubation, the sepharose beads were centrifuged and washed 4 times with ice-cold lysis buffer. The precipitates were boiled in SDS loading buffer (1×) for 10 minutes at 100°C and then analyzed by Western blotting.

### Phos-tag SDS-PAGE.

Cell pellets were resuspended in SDS loading buffer (1×) and boiled at 100°C for 10 minutes. SDS-PAGE gels supplemented with 50 μM MnCl_2_ and 50 μM Phostag AAL (Wako) were used to separate proteins. When the dye front escaped, the gels were washed for 10 minutes in transfer buffer supplemented with 1 mM EDTA. After 3 wash steps, the gels were transferred onto nitrocellulose membranes (66485, Pall), and standard Western blotting was performed to detect phosphorylated proteins.

### Statistics.

Data analysis was performed using GraphPad Prism 7.0 (GraphPad Software). Statistical analysis was performed using an unpaired *t* test, 1-way ANOVA, or 2-way ANOVA. *P* values of less than 0.05 were considered statistically significant.

### Study approval.

All animal experiments were performed according to the guidelines published by the Association for Assessment and Accreditation of Laboratory Animal Care International (AAALAC), and the animal studies were approved by the Department of Laboratory Animal Science of Fudan University (Shanghai, China).

## Authors contributions

YD, YZ, ZL, and WJ conceived and designed the study. The order of the co–first authors’ names was determined on the basis of their contributions to this study (XW and J Huang performed a majority of the experiments, and FL collected the tissue microarray samples and analyzed the related data). QY performed Western blot analyses. RW and JW performed the PD-1–binding experiments. ZZ performed a portion of the animal experiments. JY, and J Hou performed the immunohistochemistry experiments. XW and J Huang wrote the manuscript. WJ and JSS edited the manuscript. All authors read and approved the final manuscript.

## Supplementary Material

Supplemental data

## Version 1 03/16/2023

In-Press Preview

## Version 2 05/01/2023

Electronic publication

## References

[1] Barr AR, Gergely F (2007). Aurora-A: the maker and breaker of spindle poles. J Cell Sci.

[2] Liewer S, Huddleston A (2018). Alisertib: a review of pharmacokinetics, efficacy and toxicity in patients with hematologic malignancies and solid tumors. Expert Opin Investig Drugs.

[3] Durlacher CT (2016). An update on the pharmacokinetics and pharmacodynamics of alisertib, a selective Aurora kinase A inhibitor. Clin Exp Pharmacol Physiol.

[4] Bavetsias V, Linardopoulos S (2015). Aurora kinase inhibitors: current status and outlook. Front Oncol.

[5] O’Connor OA (2019). Randomized phase III study of alisertib or investigator’s choice (selected single agent) in patients with relapsed or refractory peripheral T-cell lymphoma. J Clin Oncol.

[6] Yin T (2019). Aurora A inhibition eliminates myeloid cell-mediated immunosuppression and enhances the efficacy of anti-PD-L1 therapy in breast cancer. Cancer Res.

[7] Sun S (2021). Nuclear Aurora kinase A triggers programmed death-ligand 1-mediated immune suppression by activating MYC transcription in triple-negative breast cancer. Cancer Commun (Lond).

[8] Boussiotis VA (2016). Molecular and biochemical aspects of the PD-1 checkpoint pathway. N Engl J Med.

[9] Doroshow DB (2021). PD-L1 as a biomarker of response to immune-checkpoint inhibitors. Nat Rev Clin Oncol.

[10] Kuzume A (2020). Immune-checkpoint blockade therapy in lymphoma. Int J Mol Sci.

[11] Seidel JA (2018). Anti-PD-1 and anti-CTLA-4 therapies in cancer: mechanisms of action, efficacy, and limitations. Front Oncol.

[12] Ribas A, Wolchok JD (2018). Cancer immunotherapy using checkpoint blockade. Science.

[13] Murciano-Goroff YR (2020). The future of cancer immunotherapy: microenvironment-targeting combinations. Cell Res.

[14] Sharma P (2017). Primary, adaptive, and acquired resistance to cancer immunotherapy. Cell.

[15] Wang Q, Wu X (2017). Primary and acquired resistance to PD-1/PD-L1 blockade in cancer treatment. Int Immunopharmacol.

[16] Teixidó C (2018). PD-L1 expression testing in non-small cell lung cancer. Ther Adv Med Oncol.

[17] Darvin P (2018). Immune checkpoint inhibitors: recent progress and potential biomarkers. Exp Mol Med.

[18] Ribas A, Hu-Lieskovan S (2016). What does PD-L1 positive or negative mean?. J Exp Med.

[19] Sunshine J, Taube JM (2015). PD-1/PD-L1 inhibitors. Curr Opin Pharmacol.

[20] Havel JJ (2019). The evolving landscape of biomarkers for checkpoint inhibitor immunotherapy. Nat Rev Cancer.

[21] Bontkes HJ (1997). Assessment of cytotoxic T-lymphocyte phenotype using the specific markers granzyme B and TIA-1 in cervical neoplastic lesions. Br J Cancer.

[22] Lin H (2018). Host expression of PD-L1 determines efficacy of PD-L1 pathway blockade-mediated tumor regression. J Clin Invest.

[23] Tang H (2018). PD-L1 on host cells is essential for PD-L1 blockade-mediated tumor regression. J Clin Invest.

[24] Curiel TJ (2003). Blockade of B7-H1 improves myeloid dendritic cell-mediated antitumor immunity. Nat Med.

[25] Lau J (2017). Tumour and host cell PD-L1 is required to mediate suppression of anti-tumour immunity in mice. Nat Commun.

[26] Dunphy G (2018). Non-canonical activation of the DNA sensing adaptor STING by ATM and IFI16 Mediates NF-κB signaling after nuclear DNA Damage. Mol Cell.

[27] Hu S (2012). The pharmacological NF-κB inhibitor BAY11-7082 induces cell apoptosis and inhibits the migration of human uveal melanoma cells. Int J Mol Sci.

[28] Zhang W (2020). Sustained release of TPCA-1 from silk fibroin hydrogels preserves keratocyte phenotype and promotes corneal regeneration by inhibiting interleukin-1β signaling. Adv Healthc Mater.

[29] Woo SR (2014). STING-dependent cytosolic DNA sensing mediates innate immune recognition of immunogenic tumors. Immunity.

[30] Jiang M (2020). cGAS-STING, an important pathway in cancer immunotherapy. J Hematol Oncol.

[31] Garcia-Diaz A (2017). Interferon receptor signaling pathways regulating PD-L1 and PD-L2 expression. Cell Rep.

[32] Nash P (2021). Points to consider for the treatment of immune-mediated inflammatory diseases with Janus kinase inhibitors: a consensus statement. Ann Rheum Dis.

[33] Zhang C (2019). Structural basis of STING binding with and phosphorylation by TBK1. Nature.

[34] Kemp MG (2015). UV light potentiates STING (stimulator of interferon genes)-dependent innate immune signaling through deregulation of ULK1 (Unc51-like kinase 1). J Biol Chem.

[35] Gonugunta VK (2017). Trafficking-mediated STING degradation requires sorting to acidified endolysosomes and can be targeted to enhance anti-tumor response. Cell Rep.

[36] Zhou Z (2020). Dual TBK1/IKKε inhibitor amlexanox mitigates palmitic acid-induced hepatotoxicity and lipoapoptosis in vitro. Toxicology.

[37] Banin S (1998). Enhanced phosphorylation of p53 by ATM in response to DNA damage. Science.

[38] Katayama H (2004). Phosphorylation by aurora kinase A induces Mdm2-mediated destabilization and inhibition of p53. Nat Genet.

[39] Liu Y (2021). STING, a promising target for small molecular immune modulator: A review. Eur J Med Chem.

[40] Kwon J, Bakhoum SF (2020). The cytosolic DNA-sensing cGAS-STING pathway in cancer. Cancer Discov.

[41] Yu L, Liu P (2021). Cytosolic DNA sensing by cGAS: regulation, function, and human diseases. Signal Transduct Target Ther.

[42] Kinoshita E (2006). Phosphate-binding tag, a new tool to visualize phosphorylated proteins. Mol Cell Proteomics.

[43] Deretic J (2019). A rapid computational approach identifies SPICE1 as an Aurora kinase substrate. Mol Biol Cell.

[44] Katsha A (2015). Aurora kinase A in gastrointestinal cancers: time to target. Mol Cancer.

[45] Li T (2021). Phosphorylation and chromatin tethering prevent cGAS activation during mitosis. Science.

[46] Tatsuka M (2009). Oncogenic role of nuclear accumulated Aurora-A. Mol Carcinog.

[47] Zheng F (2016). Nuclear AURKA acquires kinase-independent transactivating function to enhance breast cancer stem cell phenotype. Nat Commun.

[48] Chen T (2021). The genome sequence archive family: toward explosive data growth and diverse data types. Genomics Proteomics Bioinformatics.

[49] CNCB-NGDC Members (2022). Database Resources of the National Genomics Data Center, China National Center for Bioinformation in 2022. Nucleic Acids Res.

