# Supplementary material for: Aurora A kinase inhibition compromises its antitumor efficacy by elevating PD-L1 expression
Source: J Clin Invest. 2023 May 1;133(9):e161929. doi: 10.1172/JCI161929 (PMC10145933; doi:10.1172/JCI161929)

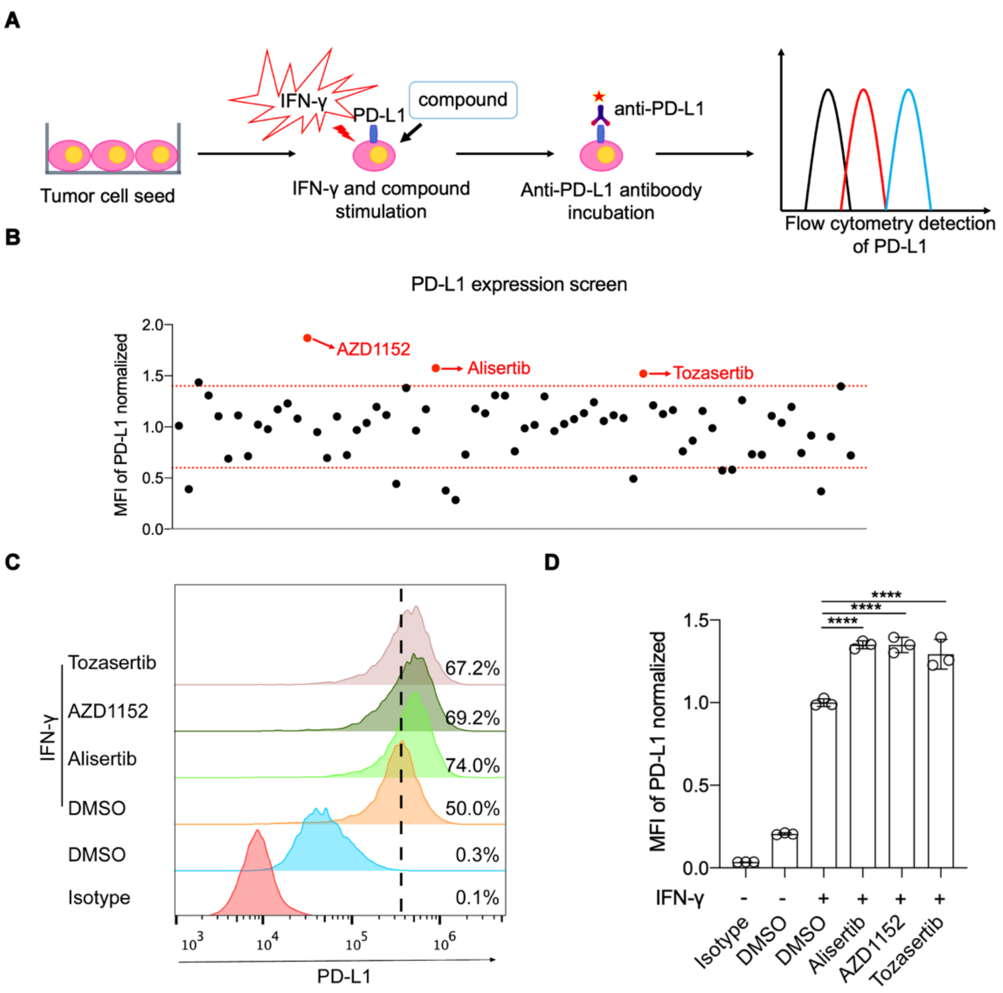

Figure S1. Aurora kinase inhibitors were identified to elevate PD-L1 expression in tumor cells. (A) Schematic of the HTFCS system. BxPC3 cells were seed to the 96-well plate, replacement the medium with complete medium containing 500 IU/mL IFN-γ and interested compound when the cells adhered to the bottom of plate, 48 hours later, cells were stained by fluorescence labelled PD-L1 antibody and detected by flow cytometry. **(B)** Results of HTFCS using a kinase inhibitor library. Highlighting by red dot were Aurora inhibitors, alisertib, AZD1152, tozasertib. **(C)** Flow cytometry analysed the cell surface PD-L1 expression in BxPC3 cells upon 1 μmol/L alisertib, 0.5 μmol/L AZD1152 or 0.5 μmol/L tozasertib treated 72 hours in the presence of 500 IU/mL IFN-γ. **(D)** The normalized mean fluorescence intensities (MFI) of (C), n=3. Data indicated mean ± standard deviation (SD). Statistical analysis was performed by one-way ANOVA. ns, no significance; *, p<0.05; **, p<0.01; ***, p<0.001; ****, p<0.0001.

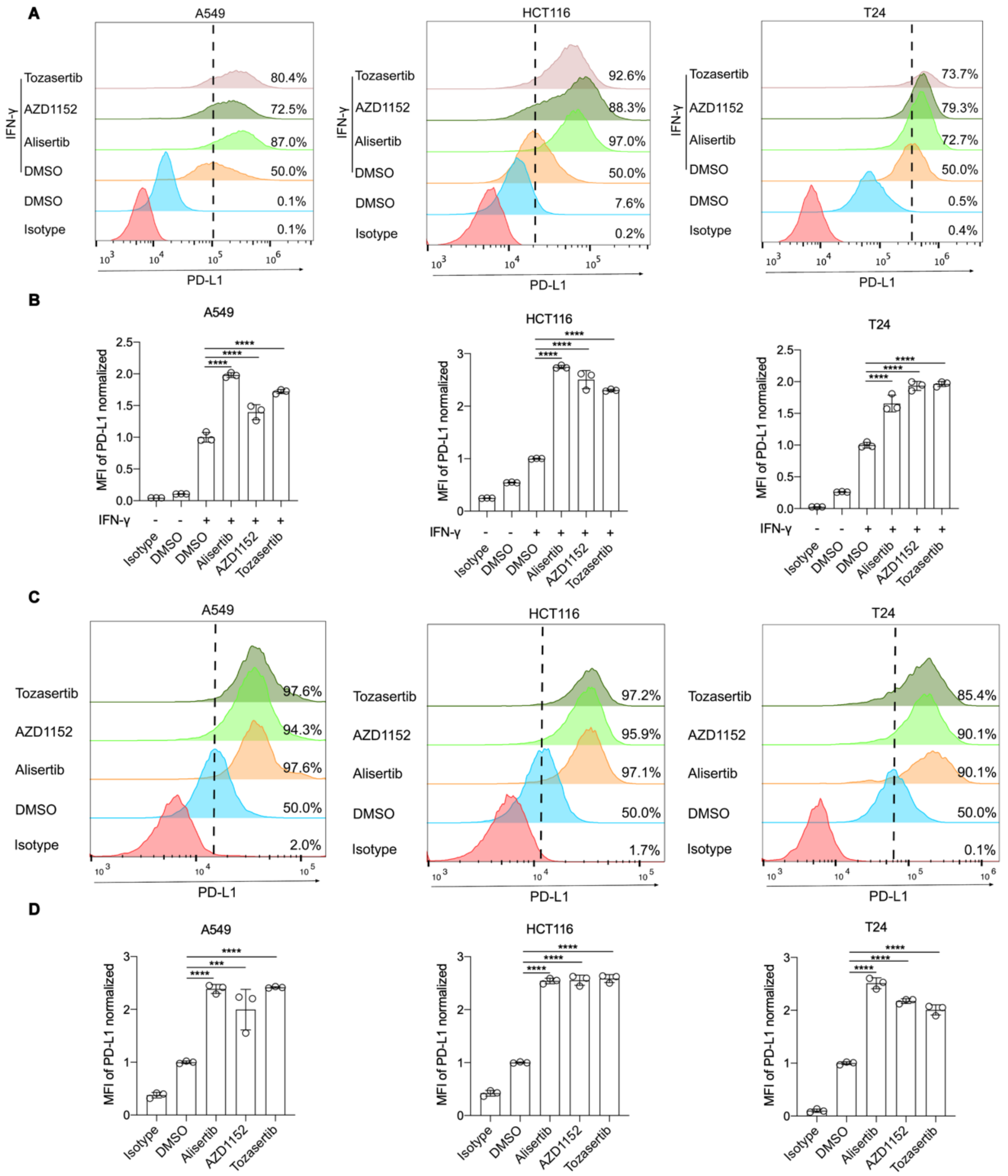

Figure S2. Aurora kinase inhibitors elevated PD-L1 expression in different types of tumor cells independent of IFN-γ. (A, C) Flow cytometry analysed the cell surface PD-L1 expression in A549, HCT116, T24 cell lines upon 1 μmol/L alisertib, 0.5 μmol/L AZD1152 or 0.5 μmol/L tozasertib treated 72 hours in the presence **(A)** or the absence **(C)** of 500 IU/mL IFN-γ. **(B, D)** The normalized mean fluorescence intensities (MFI) of (A, C), n=3. Data indicated mean ± standard deviation (SD). Statistical analysis was performed by one-way ANOVA. ns, no significance; *, p<0.05; **, p<0.01; ***, p<0.001; ****, p<0.0001.

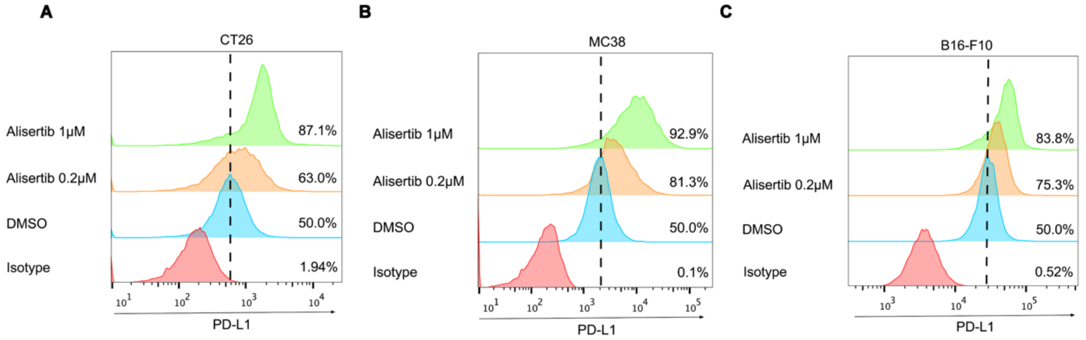

Figure S3. Alisertib elevated PD-L1 expression in murine cancer cell lines. (A-C) Flow cytometry analysed the cell surface PD-L1 expression in CT26 **(A)**, MC38 **(B)**, B16-F10 **(C)** cell lines upon alisertib treated 72 hours at the indicated concentrations.

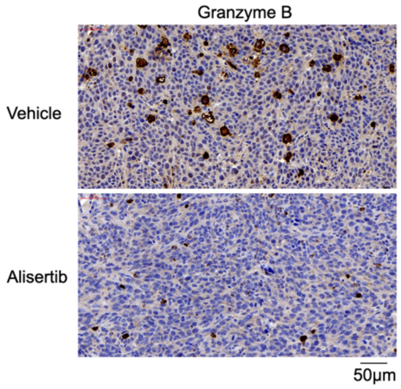

Figure S4. Immunohistochemical (IHC) staining of Granzyme B in CT26 tumor mouse models. The representative images are shown.

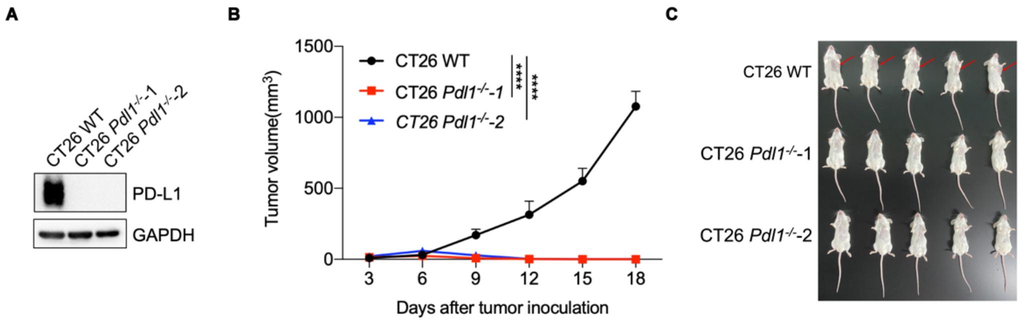

Figure S5. The growth of *Pdl1*$^{-/-}$ CT26 cells in vivo. (A) Western blot analysed the PD-L1 expression in WT or *Pdl1*$^{-/-}$ CT26 cells. **(B)** The growth curve of WT or *Pdl1*$^{-/-}$ CT26 cells in BALB/c mice, n=5. **(C)** The image of tumor bearing mice in the endpoint. The site of tumor was indicated by red lines. Data indicated mean ± SD. Two-way ANOVA was applied to compare time-dependent tumor growth. ns, no significance; *, p<0.05; **, p<0.01; ***, p<0.001; ****, p<0.0001.

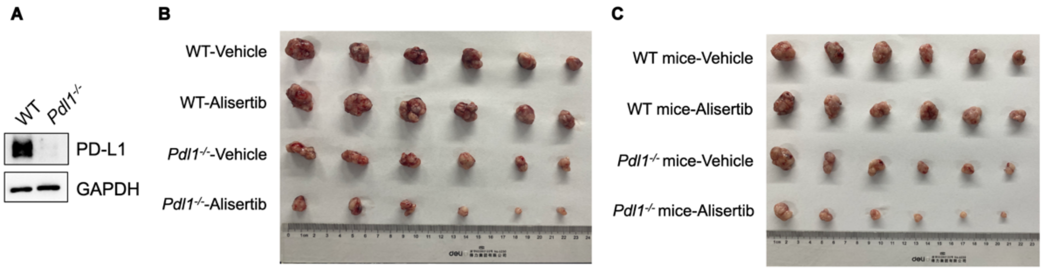

Figure S6. Both the PD-L1 in tumor cells and host immune cells compromised the antitumor efficacy of alisertib (A) Western blot analysed the PD-L1 expression in WT or *Pdl1*$^{-/-}$ MC38 cells. **(B)** Effect of vehicle or alisertib treatment on tumor growth on WT or *Pdl1*$^{-/-}$ MC38 tumor mouse models. **(C)** Effect of vehicle or alisertib treatment on MC38 growth in WT or *Pdl1*$^{-/-}$ C57BL/6 mice.

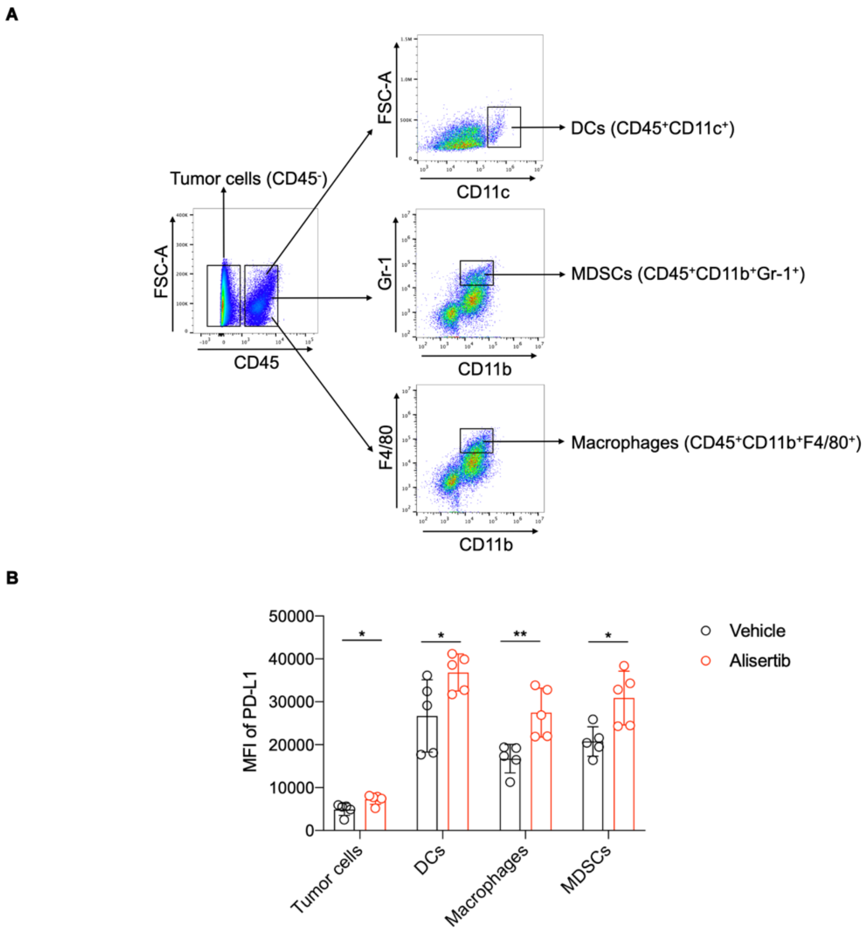

Figure S7. Alisertib upregulated the PD-L1 expression in both tumor cells and host myeloid cells. (A) Gating strategy in flow cytometry. **(B)** Flow cytometry analysed the mean fluorescence intensities of PD-L1 in tumor cells and host myeloid cells, n=5. Data indicated mean ± SD. The statistical summary was done with unpaired t test. ns, no significance; *, p<0.05; **, p<0.01; ***, p<0.001; ****, p<0.0001.

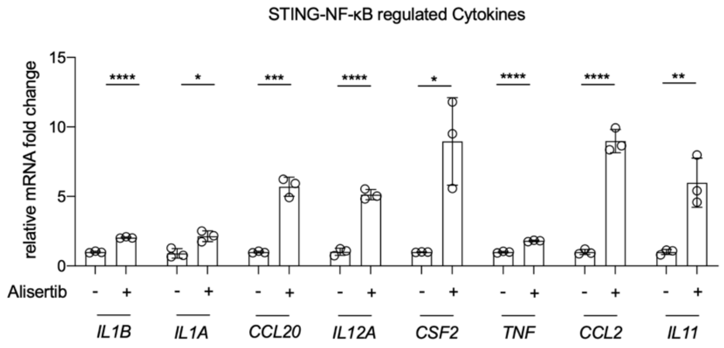

Figure S8. Alisertib increased the expression of STING-NF-κB regulated cytokines. qRT-PCR analysed the expression of indicated genes in BxPC3 cells after treated with alisertib for 72 hours, n=3. Data indicated mean ± SD. The statistical summary was done with unpaired t test. ns, no significance; *, $p<0.05$; **, $p<0.01$; ***, $p<0.001$; ****, $p<0.0001$.

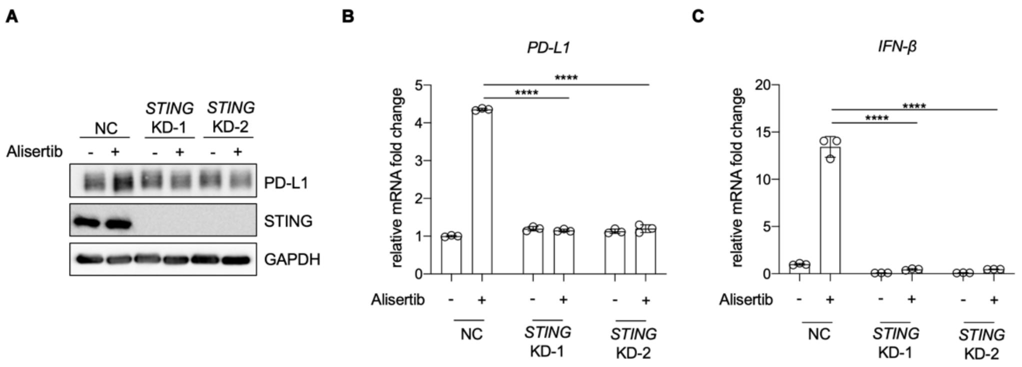

Figure S9. STING was essential for alisertib-induced PD-L1 and IFN-β increasing. (A-C) BxPC3 NC (negative control) cells or BxPC3 *STING* KD (knock down) cells were treated by 1 μmol/L alisertib for 72 hours. **(A)** Western blot analysed the indicated protein levels. qRT-PCR analysed the *PD-L1* **(B)** and *IFN-β* **(C)** mRNA levels, n=3. Data indicated mean ± SD. Statistical analysis was performed by two-way ANOVA. Ns, no significance; *, $p<0.05$; **, $p<0.01$; ***, $p<0.001$; ****, $p<0.0001$.

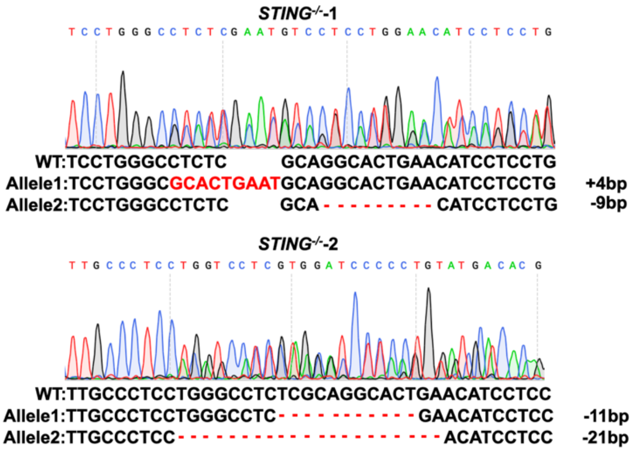

Figure S10. Sequences of the modified *STING* gene in *STING*$^{-/-}$ BxPC3 cells. The region near the *STING* guide RNA was amplified using PCR reaction, the PCR products were separated by agarose gel electrophoresis, the correct size of DNA fragment were sequenced and aligned with the WT *STING* sequences.

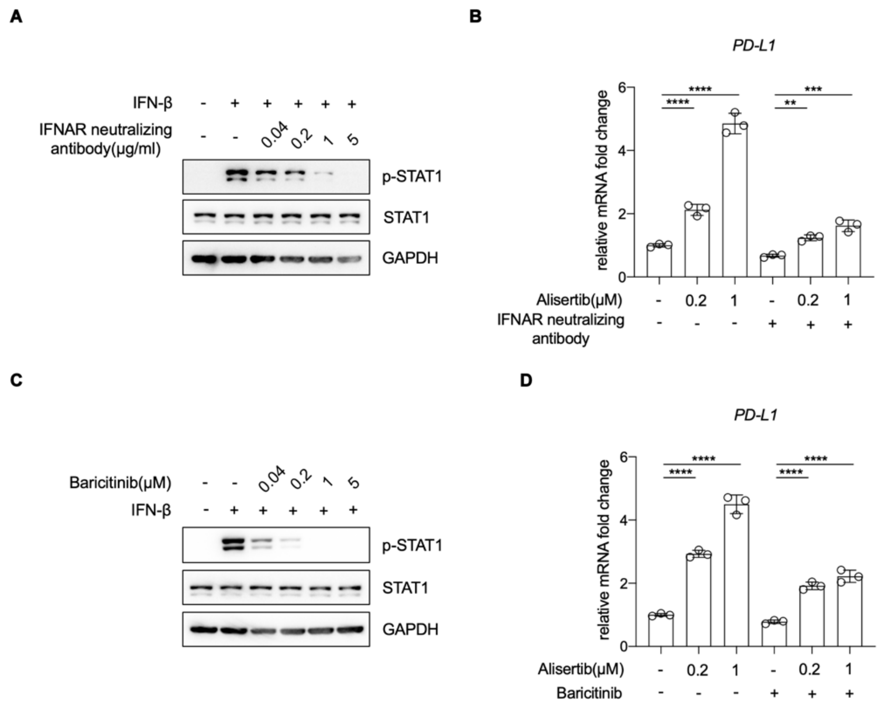

Figure S11. Alisertib could upregulate PD-L1 expression when the function of IFN-β was blocked. (A, C) BxPC3 cells were pre-treated with the indicated concentrations of IFNAR neutralizing antibody **(A)** or baricitinib **(C)** for 3 hours and followed with 10 ng/mL IFN-β for 30 minutes, western blot analysed the indicated proteins. **(B, D)** BxPC3 cells were pre-treated with the 5 μg/mL IFNAR neutralizing antibody **(B)** or 1 μmol/L baricitinib **(D)** for 3 hours and followed with 1 μmol/L alisertib for 72 hours, qRT-PCR analysed the *PD-L1* mRNA levels, n=3. Data indicated mean ± SD. Statistical analysis was performed by one-way ANOVA. ns, no significance; *, $p<0.05$; **, $p<0.01$; ***, $p<0.001$; ****, $p<0.0001$.

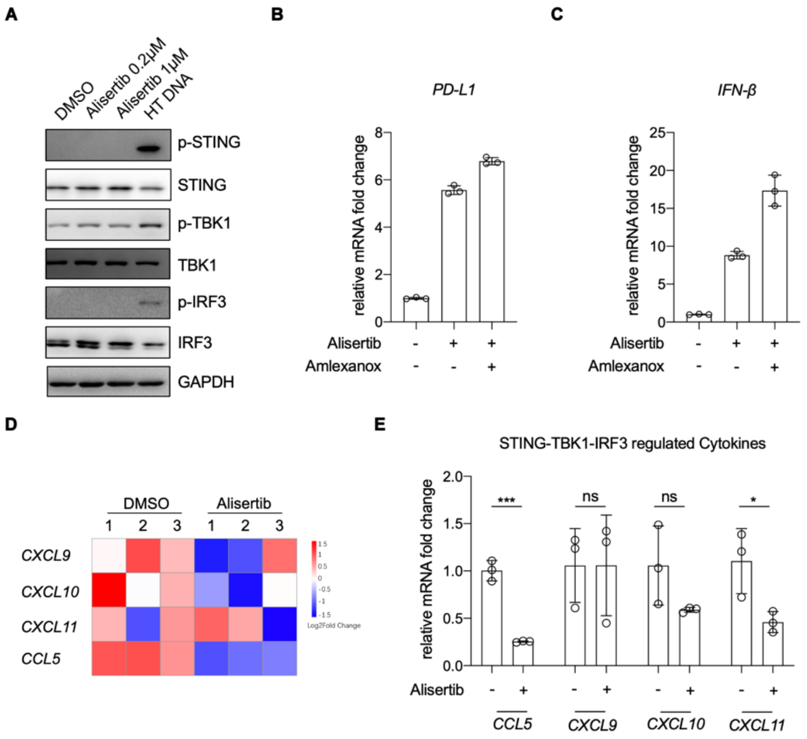

Figure S12. STING-TBK1-IRF3 pathway was irrelevant for alisertib-induced PD-L1 upregulation. (A) BxPC3 cells were treated with 0.2 μmol/L, 1 μmol/L Alisertib for 72 hours, 1 μg/mL HT-DNA were transfected to BxPC3 cells for 6 hours. Western blot analysed the indicated protein level. **(B-C)** BxPC3 cells were pre-treated with 2 μmol/L TBK1 inhibitor (Amlexanox) for 6 hours, then followed with 1 μmol/L Alisertib for 72 hours. *PD-L1* **(B)** and *IFN-β* **(C)** mRNA expression was detected by qRT-PCR, n=3. **(D)** Heat map of gene expression levels of indicated chemokines in DMSO or alisertib treated BxPC3 cells. **(E)** BxPC3 cells were treated with 1 μmol/L alisertib for 72 hours, *CCL5*, *CXCL9*, *CXCL10*, *CXCL11* mRNA levels were determined by qRT-PCR, n=3. Data indicated mean ± SD. The statistical summary was done with unpaired t test. ns, no significance; *, p<0.05; **, p<0.01; ***, p<0.001; ****, p<0.0001.

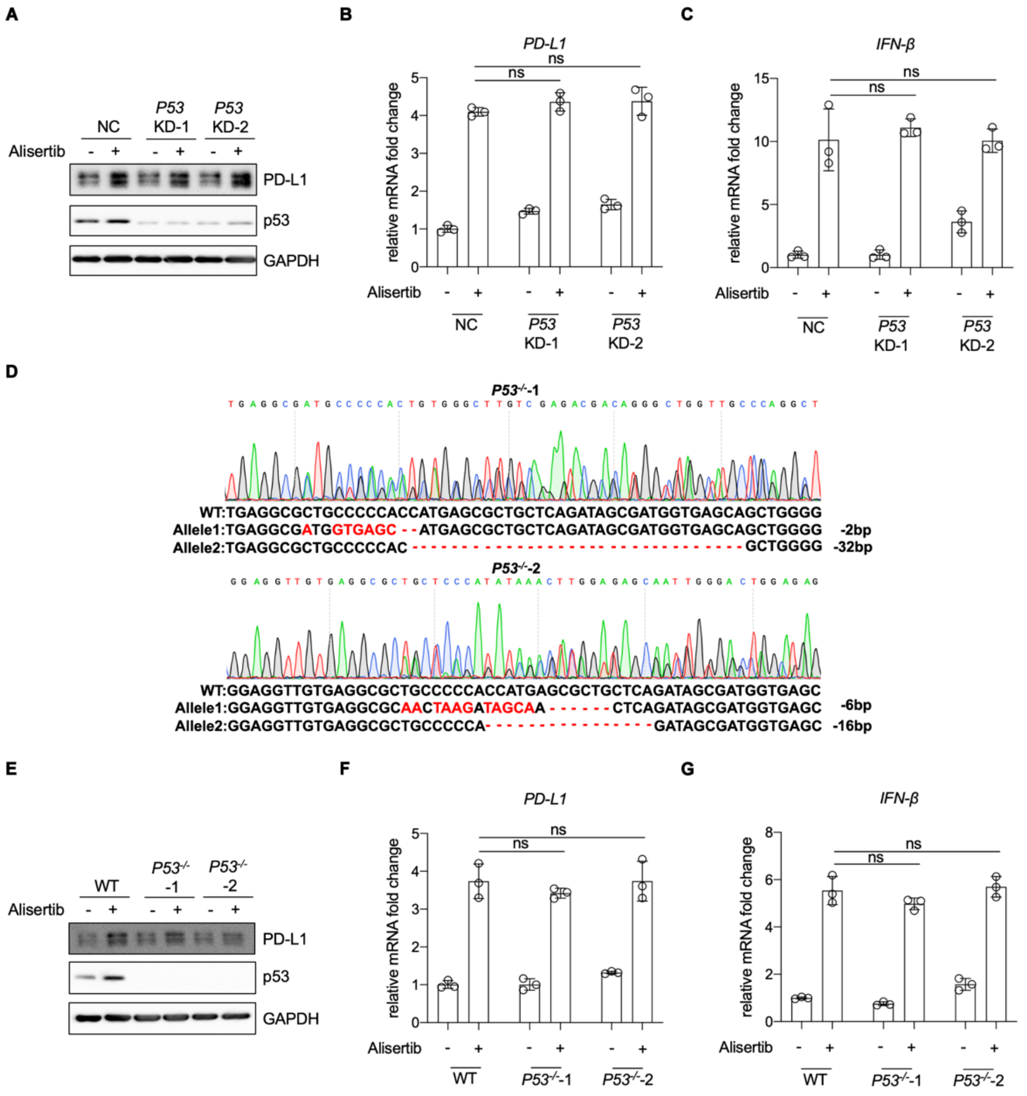

Figure S13. p53 was dispensable for alisertib-induced PD-L1 and IFN-β upregulation. (A-C) BxPC3 NC (negative control) cells or BxPC3 *P53* KD cells were treated by 1 μmol/L alisertib for 72 hours. **(A)** Western blot analysed the indicated protein levels. qRT-PCR analysed the *PD-L1* **(B)** and *IFN-β* **(C)** mRNA levels, n=3. **(D)** Sequences of the modified *P53* gene in $P53^{-/-}$ BxPC3 cells. The region near the *P53* guide RNA was amplified using PCR reaction, the PCR products were separated by agarose gel electrophoresis, the correct size of DNA fragment were sequenced and aligned with the WT *P53* sequences. **(E-G)** WT BxPC3 cells or $P53^{-/-}$ BxPC3 cells were treated by 1 μmol/L alisertib for 72 hours. **(E)** Western blot analysed the indicated protein levels. qRT-PCR analysed the *PD-L1* **(F)** and *IFN-β* **(G)** mRNA levels, n=3. Data indicated mean

± SD. Statistical analysis was performed by two-way ANOVA. ns, no significance; *, p<0.05; **, p<0.01; ***, p<0.001; ****, p<0.0001.

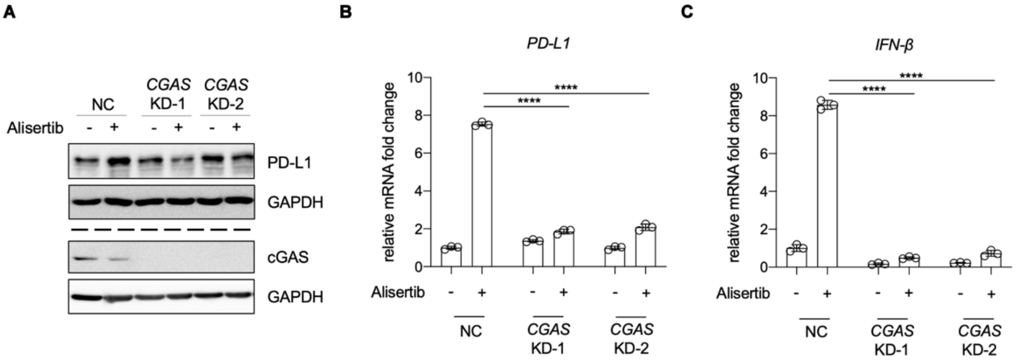

Figure S14. cGAS was essential for alisertib-induced PD-L1 and IFN-β increasing. (A-C) BxPC3 NC (negative control) cells or BxPC3 *CGAS* KD cells were treated by 1 μmol/L alisertib for 72 hours. **(A)** Western blot analysed the indicated protein levels. PD-L1 and cGAS were detected separately in two gels by using the same biological samples, and GAPDH in each gel was served as the loading control. qRT-PCR analysed the *PD-L1* **(B)** and *IFN-β* **(C)** mRNA levels, n=3. Data indicated mean ± SD. Statistical analysis was performed by two-way ANOVA. ns, no significance; *, p<0.05; **, p<0.01; ***, p<0.001; ****, p<0.0001.

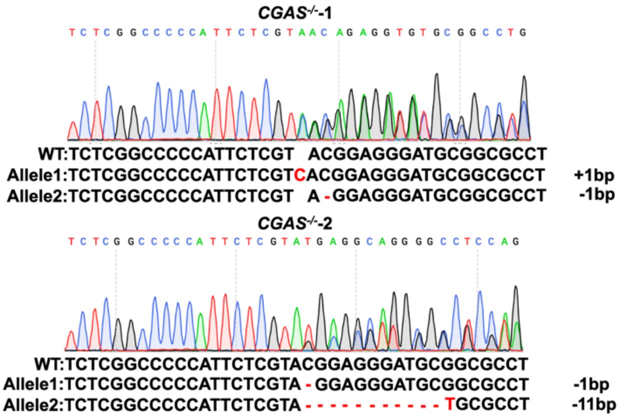

Figure S15. Sequences of the modified *CGAS* gene in *CGAS*$^{-/-}$ BxPC3 cells. The region near the *CGAS* guide RNA was amplified using PCR reaction, the PCR products were separated by agarose gel electrophoresis, the correct size of DNA fragment were

sequenced and aligned with the WT *CGAS* sequences.

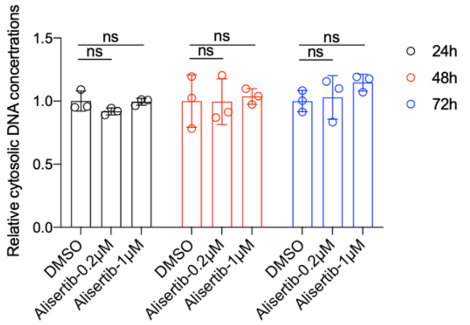

Figure S16. Alisertib did not affect the release of cytosolic DNA. BxPC3 cells were treated by 0.2/1 μmol/L alisertib for 24/48/72 hours, relative cytosolic DNA concentrations were determined by QuantiFluor dsDNA System and normalized with the protein concentrations, n=3. Data indicated mean ± SD. Statistical analysis was performed by one-way ANOVA. ns, no significance; *, p<0.05; **, p<0.01; ***, p<0.001; ****, p<0.0001.

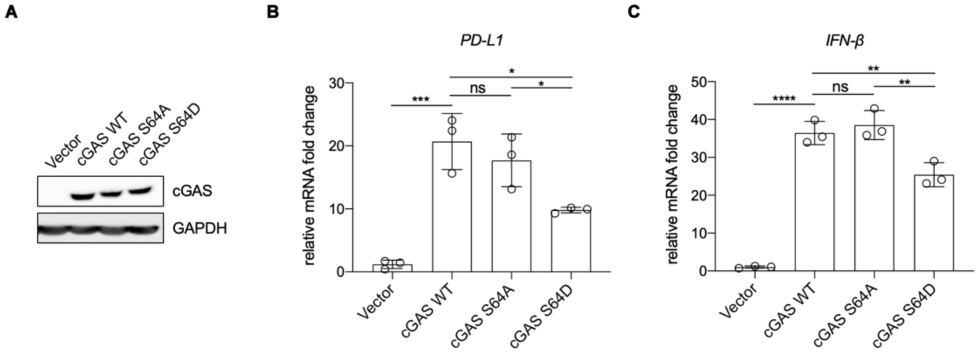

Figure S17. Phosphorylation of cGAS inhibited its activity. (A-C) cGAS WT or cGAS mutants were overexpressed to *CGAS*$^{-/-}$ BxPC3 cells. **(A)** Western blot analysed the indicated protein levels. qRT-PCR analysed the *PD-L1* **(B)** and *IFN-β* **(C)** mRNA levels, n=3. Data indicated mean ± SD. Statistical analysis was performed by one-way ANOVA. ns, no significance; *, p<0.05; **, p<0.01; ***, p<0.001; ****, p<0.0001.

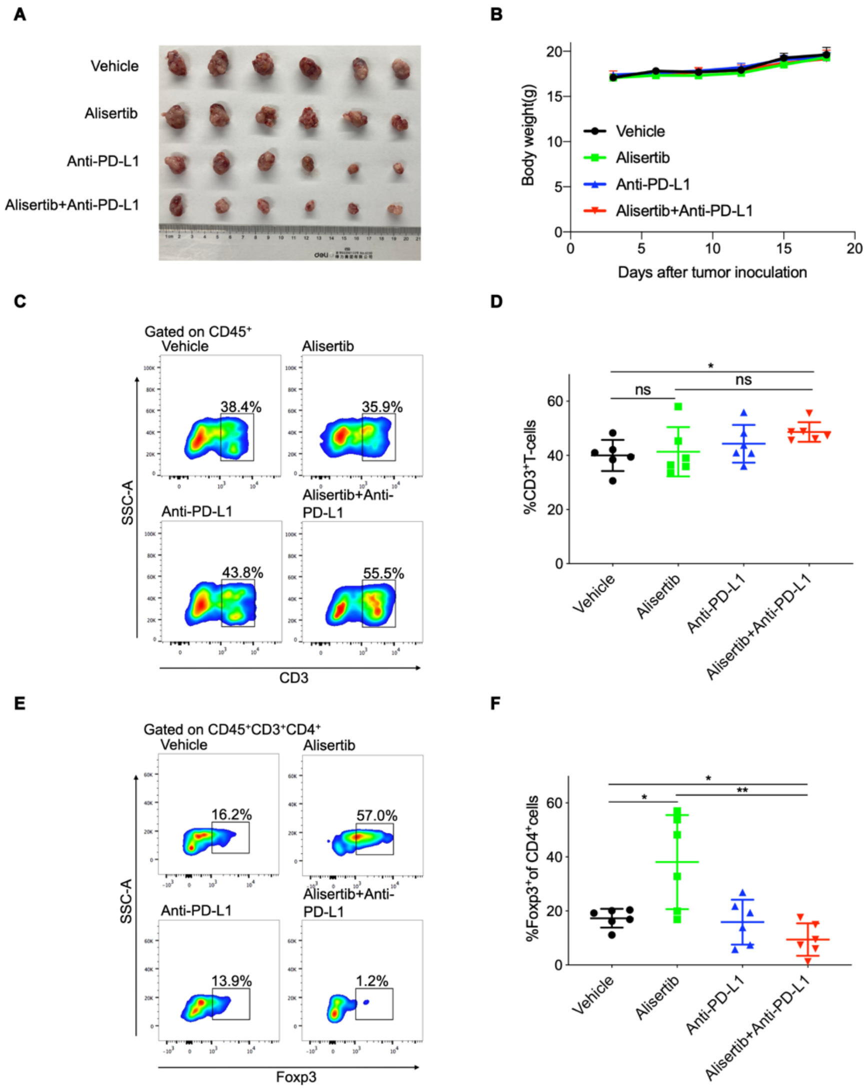

Figure S18. Anti-PD-L1 antibody improved the antitumor efficacy of alisertib. (A-F) Balb/c mice were inoculated with CT26 cells and administered with alisertib, anti-PD-L1 antibody alone or their combination. **(A)** Representative images of tumors. **(B)** body weight was measured at the indicated time points, n=6. **(C-F)** Tumor infiltrating $CD3^+$ T cells **(C-D)** and $CD4^+Foxp3^+$ Treg cells **(E-F)** were analyzed by flow cytometry, the representative plots and cumulative data for all the tumors were shown, n=6. Data indicated mean ± SD. The statistical summary was done with unpaired t test. ns, no significance; *, $p<0.05$; **, $p<0.01$; ***, $p<0.001$; ****, $p<0.0001$.

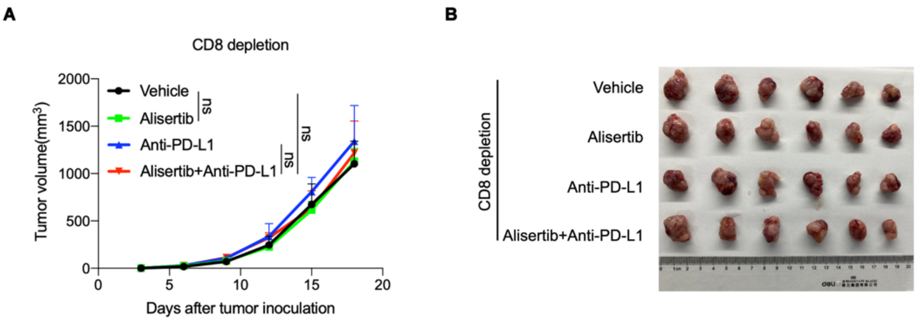

Figure S19. CD8^{+} T cells were indispensable for the combination antitumor effect of alisertib and anti-PD-L1 antibody. (A-B) BALB/c mice were inoculated with CT26 cells and administered with alisertib, anti-PD-L1 antibody alone or their combination in the presence of anti-CD8 antibody, n=6. Data indicated mean ± SD. Two-way ANOVA was applied to compare time-dependent tumor growth. ns, no significance; *, p<0.05; **, p<0.01; ***, p<0.001; ****, p<0.0001.

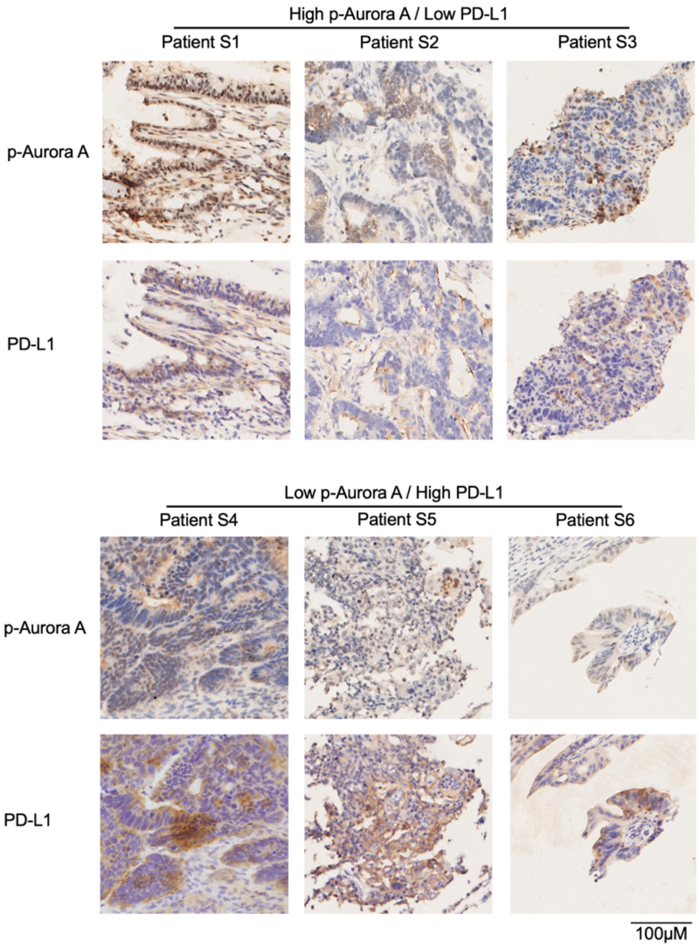

Figure S20. The levels of an active form of Aurora A (p-Aurora A T288) were negatively associated with PD-L1 expression in human tumor tissues. Representative images of IHC staining of p-Aurora A and PD-L1 in human colon cancer tissues.

Table S1. Result of high throughput screening of the kinase inhibitors library's 69 compounds. MFI was normalized to IFN-γ-DMSO treated group.

	compound	target	MFI of PD-L1 normalized
	DMSO		0.254
IFN-γ	DMSO		1.000
	DB07268	GADD45β, MKK7	1.011
	Cobimetinib	MEK	0.391
	Palbociclib	CDK	1.435
	PF-06463922	ALK, ROS1	1.308
	GSK2636771	PI3Kβ	1.105
	GDC-0994	ERK1, ERK2	0.689
	Sorafenib	Raf-1, B-Raf, VEGFR-3	1.113
	Gefitinib	EGFR	0.715
	BYL-719	PI3Kα	1.023
	SAR-20347	TYK2, JAK1, JAK2, JAK3	0.978
	GDC-0068	Akt1, Akt2, Akt3	1.171
	CHIR-99021	GSK-3α/β	1.229
	LDK378	ALK	1.080
	AZD1152	Aurora B	1.868
	Erlotinib	EGFR	0.950
	CYT387	JAK1, JAK2	0.696
	RO4929097	γ secretase	1.101
	Duvelisib	p100δ	0.724
	Solcitinib	JAK1	0.969
	CCT128930	Akt2	1.039
	Miltefosine	Akt	1.196
	Pyrimethamine	DHFR	1.116
	Tofacitinib	JAK3	0.442
	Cabozantinib	VEGFR2, c-Met, Kit, Axl, Flt3	1.382
	Sunitinib	VEGFR2, PDGFRβ	0.965
	Vemurafenib	B-RAF	1.173
	Alisertib	Aurora A	1.574

	AZD-1480	JAK2	0.378
	Ruxolitinib	JAK1, JAK2	0.284
	Dacomitinib	EGFR, ERBB2, ERBB4	0.730
	STF-31	GLUT1	1.178
	MK 2206 (dihydrochloride)	Akt1, Akt2, Akt3	1.132
	Vismodegib	hedgehog	1.310
	Everolimus	mTOR	1.305
	PCI-32765	Btk	0.760
	Y-27632	ROCK-I, ROCK-II	0.986
	Navitoclax	Bcl-2	1.020
	Temsirolimus	mTOR	1.298
	Regorafenib	VEGFR1/2/3, PDGFRβ, Kit, RET, Raf-1	0.958
	CC-930	JNK1, JNK2, JNK3	1.029
	JNK-IN-8	JNK1, JNK2, JNK3	1.076
	BEZ235	p110α/γ/δ/β, mTOR	1.133
	Rapamycin	mTOR	1.241
	RAF265	Raf	1.057
	GDC-0084	PI3Kαβδγ, mTOR	1.115
	Veliparib	PARP1, PARP2	1.086
	TG-101348	JAK2	0.493
	Tozasertib	Aurora-A, Aurora-B, Aurora-C	1.520
	AX-15836	ERK5	1.211
	Linsitinib	IGF-1R	1.126
	Brigatinib	ALK	1.165
	Vandetanib	VEGFR2	0.760
	A-443654	Akt	0.865
	Afuresertib	Akt	1.156
	BAY1125976	Akt1, Akt2	0.987
	BI 2536	Plk1	0.720
	SCH772984	ERK1, ERK2	0.573

	AS703026	MEK1, MEK2	0.582
	GSK269962A	ROCK1, ROCK2	1.261
	Volasertib	PLK1	0.733
	Saracatinib	Src	0.727
	GSK 650394	SGK	1.107
	AICAR	AMPK	1.041
	Crizotinib	c-Met, ALK	1.196
	SHP099	SHP2	0.744
	Decernotinib	JAK3	0.917
	Trametinib	MEK	0.369
	AXL1717	IGF-1R	0.904
	LY2603618	Chk1	1.396

Table S2. Summary of p-Aurora A and PD-L1 expression in the tissue microarrays.

The percentages of indicated cohorts in carcinoma.

**Carcinoma**		**p-Aurora A**			
		**Negative**	**Low**	**High**	**Total**
**PD-L1**	**Negative**	317(64.2%)	96(19.4%)	18(3.6%)	431(87.2%)
	**Low**	28(5.7%)	7(1.4%)	5(1.0%)	40(8.1%)
	**High**	10(2.0%)	12(2.5%)	1(0.2%)	23(4.7%)
	**Total**	355(71.9%)	115(23.3%)	24(4.8%)	494(100.0%)

Table S3. Summary of p-Aurora A and PD-L1 expression in the tissue microarrays.

The percentages of indicated cohorts in Para-carcinoma.

**Para-carcinoma**		**p-Aurora A**			
		**Negative**	**Low**	**High**	**Total**
**PD-L1**	**Negative**	397(80.4%)	37(7.5%)	36(7.3%)	470(95.2%)
	**Low**	9(1.8%)	1(0.2%)	2(0.4%)	12(2.4%)
	**High**	8(1.6%)	2(0.4%)	2(0.4%)	12(2.4%)
	**Total**	414(83.8%)	40(8.1%)	40(8.1%)	494(100.0%)

Table S4. Correlation analysis between p-Aurora A and PD-L1 in human colon cancer tissues. *P* value were calculated using Pearson χ^2 test.

		**p-Aurora A**			
		**Low**	**High**	**Total**	***P* value**
**PD-L1**	**Low**	7(28.0%)	5(20.0%)	12(48.0%)	
	**High**	12(48.0%)	1(4.0%)	13(52.0%)	
	**Total**	19(76.0%)	6(24.0%)	25(100.0%)	*P*=0.04

Table S5. Primers used in the knockdown and knockout study

Gene	Primer-Forward (5'-3')	Primer-Reverse (5'-3')
STING#1	CACCGGGATGTTCAGTGCCTGCGAG	AAACCTCGCAGGCACTGAACATCCC
STING#2	CACCGGCAGGCACTGAACATCCTCC	AAACGGAGGATGTTCAGTGCCTGCC
CGAS#1	CACCGCGGCCCCCATTCTCGTACGG	AAACCCGTACGAGAATGGGGGCCGC
CGAS#2	CACCGGGCCCCCATTCTCGTACGGA	AAACTCCGTACGAGAATGGGGGCCC
P53#1	CACCGTATCTGAGCAGCGCTCATGG	AAACCCATGAGCGCTGCTCAGATAC
P53#2	CACCGATCTGAGCAGCGCTCATGGT	AAACACCATGAGCGCTGCTCAGATC
Pdl1	CACCGGTATGGCAGCAACGTCACGA	AAACTCGTGACGTTGCTGCCATACC

Table S6. Primers used in the qRT-PCR study

Gene	Primer-Forward (5'-3')	Primer-Reverse (5'-3')
PD-L1	GGTGCCGACTACAAGCGAAT	AGCCCTCAGCCTGACATGTC
IFN-β	ACACTGGTCGTGTTGTTGAC	GGAAAGAGCTGTCGTGGAGA
IL1A	TGGTAGTAGCAACCAACGGGA	ACTTTGATTGAGGGCGTCATTC
IL1B	ATGATGGCTTATTACAGTGGCAA	GTCGGAGATTCGTAGCTGGA
CCL20	AACCATGTGCTGTACCAAGAGT	AAGTTGCTTGCTTCTGATTCGC
IL12A	CCTTGCACTTCTGAAGAGATTGA	ACAGGGCCATCATAAAAGAGGT
CSF2	TCCTGAACCTGAGTAGAGACAC	TGCTGCTTGTAGTGGCTGG
TNF	CCTCTCTCTAATCAGCCCTCTG	GAGGACCTGGGAGTAGATGAG
CCL2	CAGCCAGATGCAATCAATGCC	TGGAATCCTGAACCCACTTCT
IL11	CGAGCGGACCTACTGTCCTA	GCCCAGTCAAGTGTCAGGTG
CCL5	CAGCAGTCGTCTTTGTCACC	AGGACTCTCCATCCTAGCTCA
CXCL9	CCAATACAGGAGTGACTTGGAAC	TCACTACTGGGTTCCTTGC
CXCL10	CTGAGCCTACAGCAGAGGAA	GAGAGGTACTCCTTGAATGCC
CXCL11	AGCAAGCAAGGCTTATAATCAAAAA	TTGTCATTTCAGTAGTCACAGTTA
GAPDH	ACCCAGAAGACTGTGGATGG	TTCAGCTCAGGGATGACCTT

Supplement materials and methods

Cell culture and transfection

The BxPC3, A549, T24, and CT26 cell lines (purchased from ATCC) were maintained in RPMI 1640 Medium (#C11875500BT, Gibco, Thermo Fisher); HEK293T, B16-F10, and HCT116 cell lines (gift from Dengke K. Ma's Lab in California University, San Francisco) were maintained in Dulbecco's Modified Eagle Medium (#C11995500BT, Gibco, Thermo Fisher); and the MC38 cell line (a gift from Jun O. Liu's Lab in Johns Hopkins University) was maintained in RPMI 1640 (ATCC modification) Medium (#A10491-01, Gibco, Thermo Fisher). All media were supplemented with 10% fetal bovine serum and 1% penicillin/streptomycin, and cells were grown in a 37°C incubator with 5% CO_2. Plasmid transfection in the HEK293T cell line was performed using PEI transfection reagent, and siRNA transfection was performed with Lipofectamine RNAiMAX Reagent (#13778-075, Invitrogen). The siRNA sequences that specifically targeted *AURKA* were #1 (5'-CUCUAUAAACUGUUCCAAGUGGUGCAU-3') and #2 (5'-GCACAAUUCUCGUGGCUACUUUCACUU-3'). The control siRNA sequence was 5'-UUCUCCGAACGUGUCACGUTT-3'. Herring testis (HT) DNA (Sigma) was transfected with Lipofectamine 2000 (Thermo Fisher).

Antibodies and reagents

All inhibitors, including alisertib, AZD1152, tozasertib, BAY11-7082, TPCA-1, amlexanox, baricitinib, and Ro-3306, were purchased from MCE (MedChemExpress). IFN-β was purchased from GenScript (Z03109). The following antibodies were used for flow cytometry, western blotting or immunohistochemistry: anti-human PD-L1 for flow cytometry (329708, clone 29E.2A3, Biolegend) or western blotting (A19135, clone ARC2478, ABclonal) and immunohistochemistry (13684, clone E1L3N, Cell Signaling Technology); anti-mouse PD-L1 for flow cytometry (564716, clone MIH5, BD Horizon) or western blotting (ab213480, clone EPR20529, Abcam); anti-IFNAR (21385-1, clone MMHAR-2, PBL Assay Science); anti-Aurora A (14475, clone D3E4Q, Cell Signaling Technology); anti-Aurora A (p Thr288) (NB100-2371, polyclonal antibody, NOVUS); anti-CD45 (557659, clone 30-F11, BD Horizon); anti-CD45 (103133, clone 30-F11,

Biolegend); anti-CD11b (562950, clone M1/70, BD Horizon); anti-CD11c (564079, clone HL3, BD Horizon); anti-Gr-1 (557979, clone RB6-8C5, BD Horizon); anti-F4/80 (123114, clone BM8, Biolegend); anti-CD3 (553061, clone 145-2C11, BD Horizon); anti-CD4 (563151, clone RM4-5, BD Horizon); anti-CD8 (551162, clone 53-6.7, BD Horizon); anti-Foxp3 (560408, clone MF23, BD Horizon); anti-Granzyme B (515408, clone GB11, Biolegend) for flow cytometry or immunohistochemistry (ab4059, polyclonal antibody, Abcam); anti-p65 (6956, clone L8F6, Cell Signaling Technology); anti-p-p65 (3033, clone 93H1, Cell Signaling Technology); anti-p53 (9282, polyclonal antibody, Cell Signaling Technology); anti-STING (13647, clone D2P2F, Cell Signaling Technology); anti-p-STING (19781, clone D7C3S, Cell Signaling Technology); anti-TBK1 (3504, clone D1B4, Cell Signaling Technology); anti-p-TBK1 (5483, clone D52C2, Cell Signaling Technology); anti-IRF3 (11904, clone D6I4C, Cell Signaling Technology); anti-p-IRF3 (4947, clone 4D4G, Cell Signaling Technology); anti-cGAS (15102, clone D1D3G, Cell Signaling Technology); anti-HA (0906-1, polyclonal antibody, HUABIO); anti-Flag (F1804, clone M2, Sigma-Aldrich); anti-GAPDH (60004-1-Ig, clone 1E6D9, Proteintech). Secondary antibodies conjugated with HRP were purchased from Jackson ImmunoResearch.

High-throughput flow cytometry screening system

BxPC3 cells were seeded in a 96-well plate with a Multidrop Combi robotic dispenser (Thermo Fisher). After incubation at 37°C overnight, cells were treated with the compound and 500 IU/mL IFNγ (#TL-105, Beijing T&L Biotechnology) for 48 hours. Cells were then digested into single cells and incubated with APC-conjugated anti-human CD274 antibodies (#329708, Biolegend) at room temperature for 30 minutes. Samples were analyzed on the IntelliCyt iQue Screener PLUS (Sartorius).

Flow Cytometry

Cells were suspended in Stain Buffer (#554657, BD Pharmingen) and incubated with primary conjugated antibodies at room temperature for 30 minutes. After washing in Stain Buffer, samples were analyzed with a BD LSRFortessa cell analyzer (BD Bioscience, USA).

Western Blotting

Cells were seeded in six-well plates and treated as described in the figure legends with the addition of SDS loading buffer (6x) and boiled at 100°C for 10 minutes. Cell lysates were subjected to SDS-PAGE and transferred to a nitrocellulose membrane (#66485, Pall). The membrane was blocked with 5% non-fat milk at room temperature for 1 hour after protein transfer. After incubation with the primary antibody overnight, the membrane was washed with PBS-T and incubated with HRP-conjugated antibody in 5% non-fat milk for 1 hour at room temperature. Finally, ECL substrate was used for exposure. The images of full, uncut gels were shown in online Supplemental Material.

Plasmid construction

P8400-IL2-exPD-1-hFc was obtained by cloning IL-2 signal peptide sequence (ATGTACAGGATGCAACTCCTGTCTTGCATTGCACTAAGTCTTGCACTTGTCACGAATTCG) into the P8400-hFc backbone at the SalI and EcoRV sites, then inserting the extracellular domain sequence (amino acid 34-150) at the EcoRV and BspEI sites. The sgRNA expression plasmid was constructed by phosphorylating and annealing each pair of complementary oligonucleotides that targeted a specific gene using T4 PNK (#M0201S, New England Biolabs), then ligating the resulting double-stranded DNA product into a BsmBI-digested lentiCRISPR V2 plasmid backbone using T4 Quick Ligase (Thermo Fisher). The complementary oligonucleotides for each target gene were shown in Table S5.

Recombinant human PD-1-Fc protein purification

Plasmid P8400-IL2-exPD-1-hFc was transfected into HEK293T cells using PEI transfection reagent, and the supernatant was collected after 72 hours. It was then lightly centrifuged, and debris of cells were filtered using a 0.45-μm filter. Protein A Resin (#L00210, GenScript) was pre-equilibrated with phosphate-buffered saline (PBS) and then incubated with the supernatant for 2 hours. The resin was washed with PBS (pH 5.0) to remove non-specifically bound protein. The protein was then eluted with 100 mM

glycine (pH 2.8) and immediately neutralized with 1 M Tris-HCl (pH 8.0). The protein was then concentrated and exchanged into PBS (pH 7.4) using an Amicon Ultra-15 Centrifugal Filters (#UFC901096, Sigma-Aldrich). Protein concentration was determined with the Pierce BCA Protein Assay Kit (#23225, Thermo Scientific) according to the manufacturer’s instructions.

PD-1 binding assay

BxPC3 cells (1×10^6) were incubated with 50 μg/mL recombinant human PD-1-Fc protein at room temperature for 30 minutes. After washing in Stain Buffer, cells were incubated with FITC-conjugated anti-human IgG antibody (#97224, Abcam) at room temperature for 30 minutes. Samples were analyzed after washing in Stain Buffer. Data were analyzed using FlowJo software.

Immunohistochemistry

For immunohistochemistry, human colon tumor tissue microarrays were obtained from Zhongshan Hospital, Fudan University, and mouse tumor tissues were obtained from tumor xenografts. In brief, sections were deparaffinized, and antigens were restored in Sodium citrate antigen retrieval solution (pH 6.0). Endogenous peroxidases were blocked in 3% H_2O_2 solution. Tissue samples were incubated with specific primary antibodies and an HRP-labeled secondary antibody and then developed using DAB reagent (Servicebio, Wuhan). Nuclei were counterstained with Hematoxylin staining solution. The histological staining results were scored according to the staining intensity and classified into three grades: negative, low, and high.

RNA extraction and quantitative Real Time PCR

Total RNA extraction reagent (Vazyme) was used to isolate total RNA from cells. cDNA was obtained by reverse transcription using HiScript II Q RT SuperMix (Vazyme) according to the manufacturer's protocol. Gene expression was measured by quantitative RT-PCR on the Quant Studio 6 Flex Real-Time PCR system (ABI). Expression of target

genes was normalized to that of GAPDH and calculated using the ΔΔCt method. The primers for each target gene were shown in Table S6.

Knockdown and knockout

Viral material was packaged by transfecting packaging plasmids PAX2, PGMD2G, and sgRNA expression plasmids with a specific target to each gene to 293T cells. After 8 hours, the medium was replaced with DMEM containing 10% FBS. The supernatant was collected after transfecting for 48 hours and 72 hours. The two supernatants were then mixed, lightly centrifuged, and filtered using a 0.45-μm filter. The viruses were then added to the target cells with 1 μg/ml polybrene. After 48 hours, the medium was completely replaced with medium containing 1 μg/ml puromycin. After 72 hours of puromycin selection, the resistant cells were harvested as knockdown cells. For the knockout cells, puromycin-resistant cells were diluted for colony formation. After the single colony had grown to an appropriate size, genomic DNA was extracted. The region near the guide RNA was amplified by PCR, the PCR products were separated by agarose gel electrophoresis, and DNA fragments of the correct size were collected and sequenced. The efficacy of knockdown and knockout were confirmed by western blotting.

Cytosolic DNA quantification

The concertrations of cytosolic DNA in cell lysate was detected by QuantiFluor dsDNA Sample Kit (E2671, Promega) according to the manufacturer's recommended procedure. Next, the results were normalized by their protein concertrations to calculate the relative cytosolic DNA concertrations.

Full unedited gel for Figure 1C

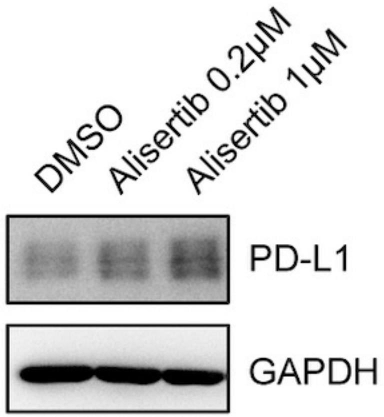

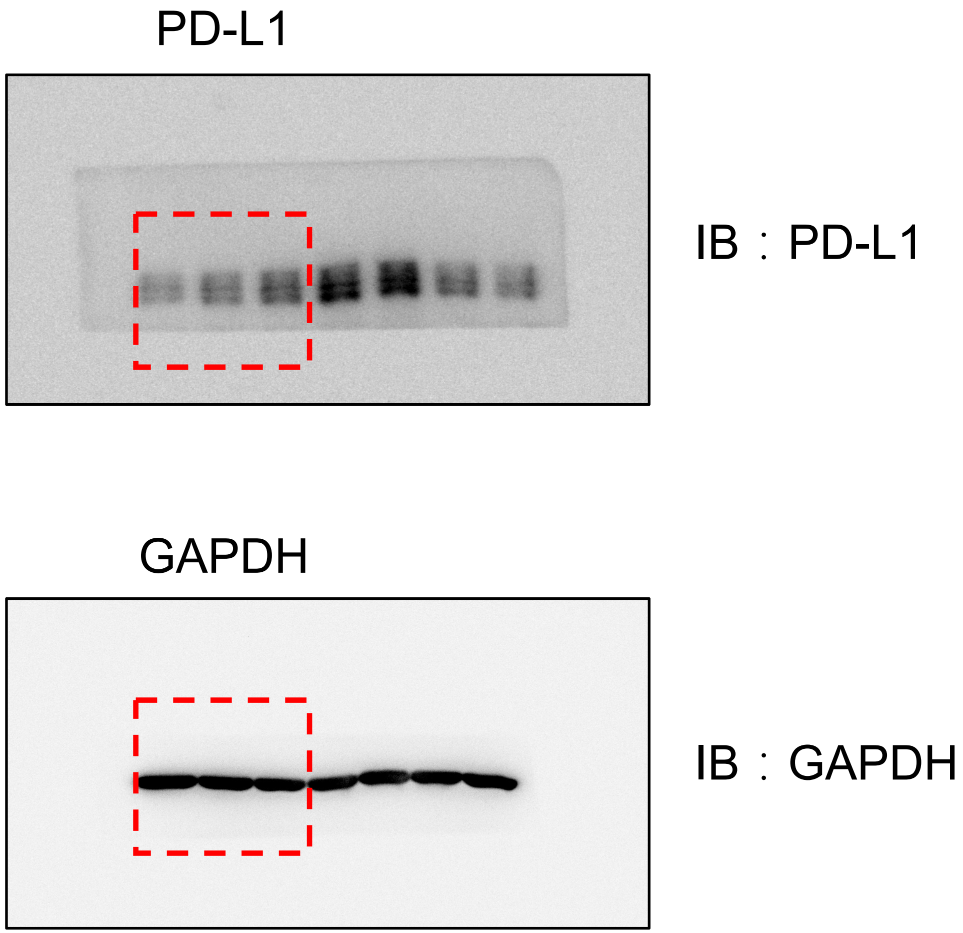

Full unedited gel for Figure 2A

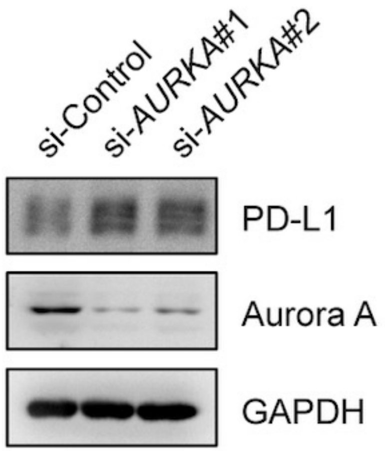

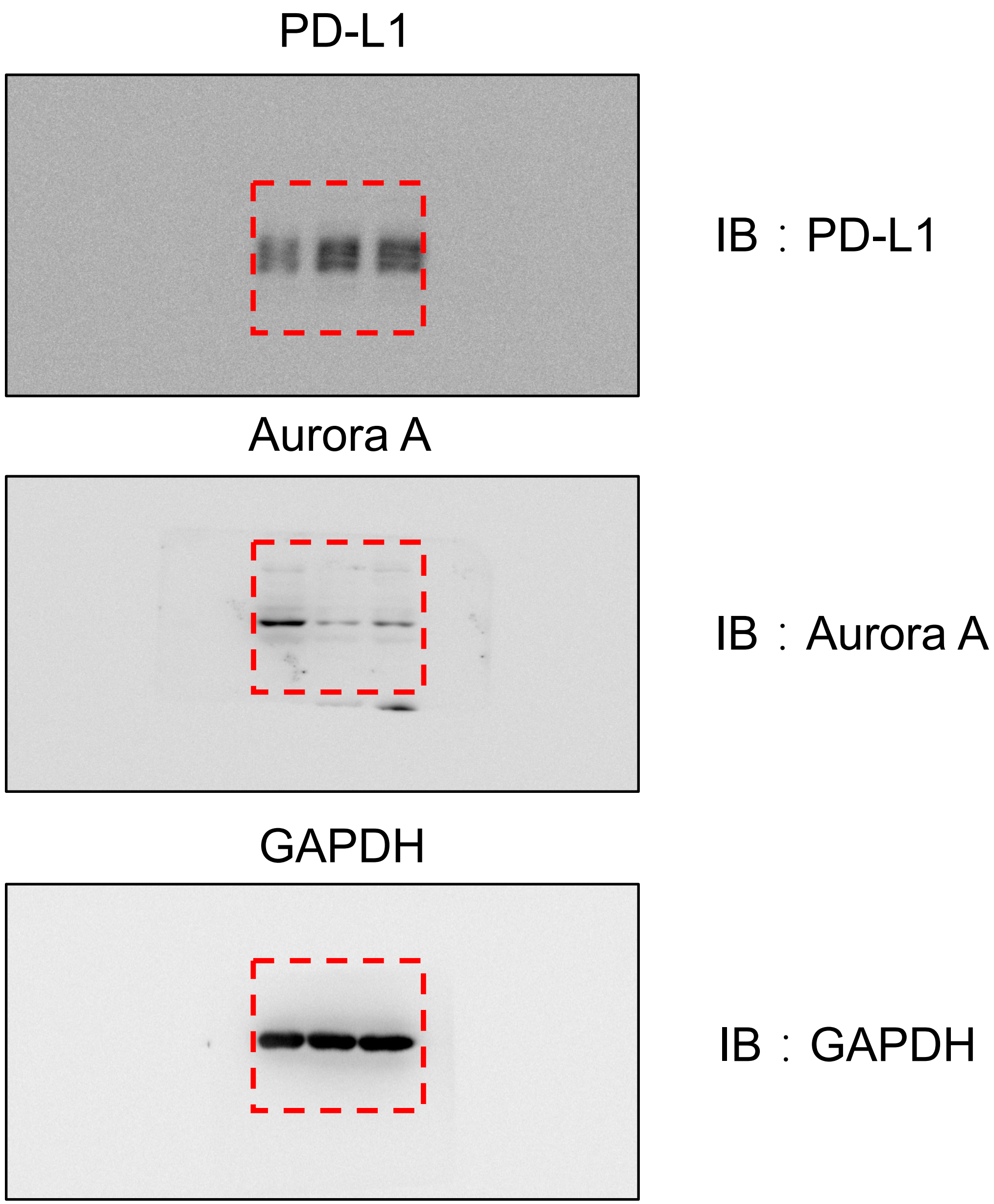

Full unedited gel for Figure 4C

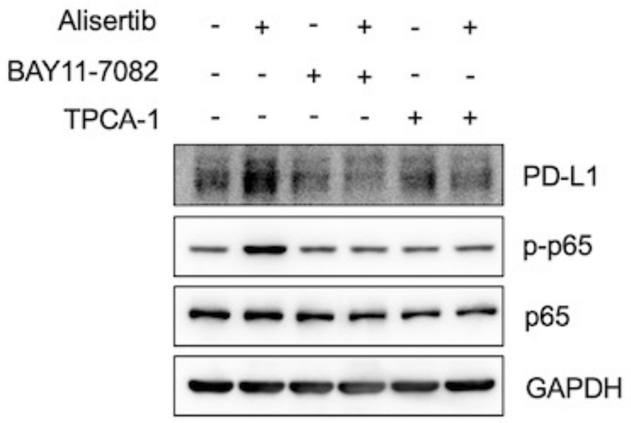

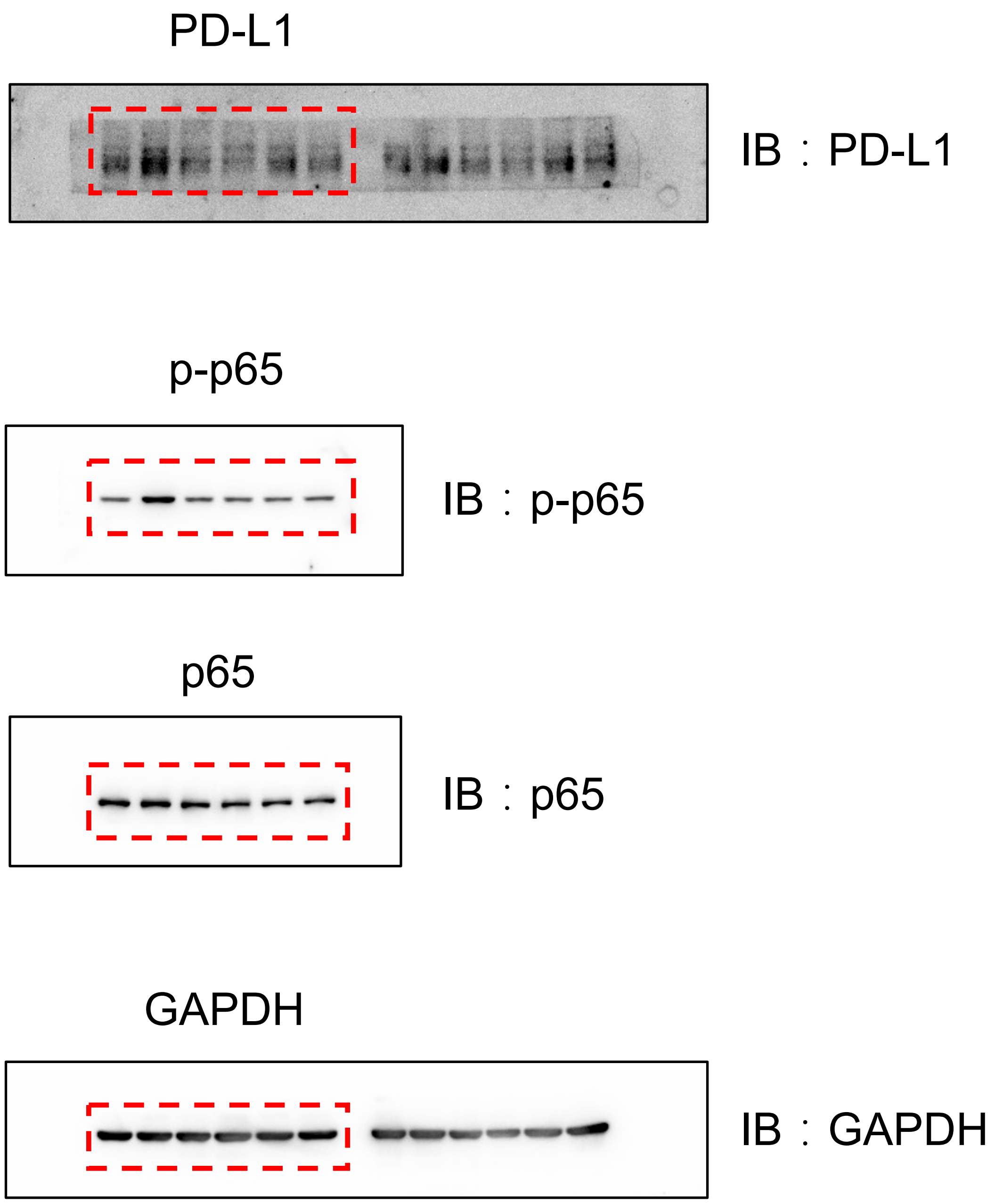

Full unedited gel for Figure 4I

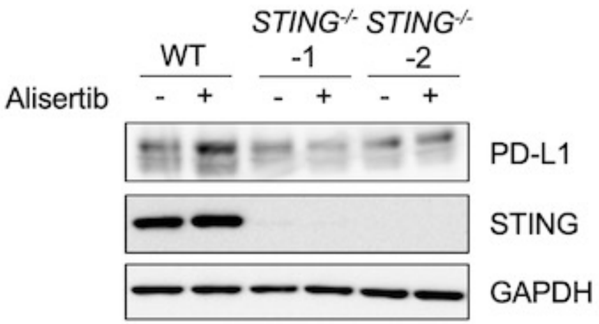

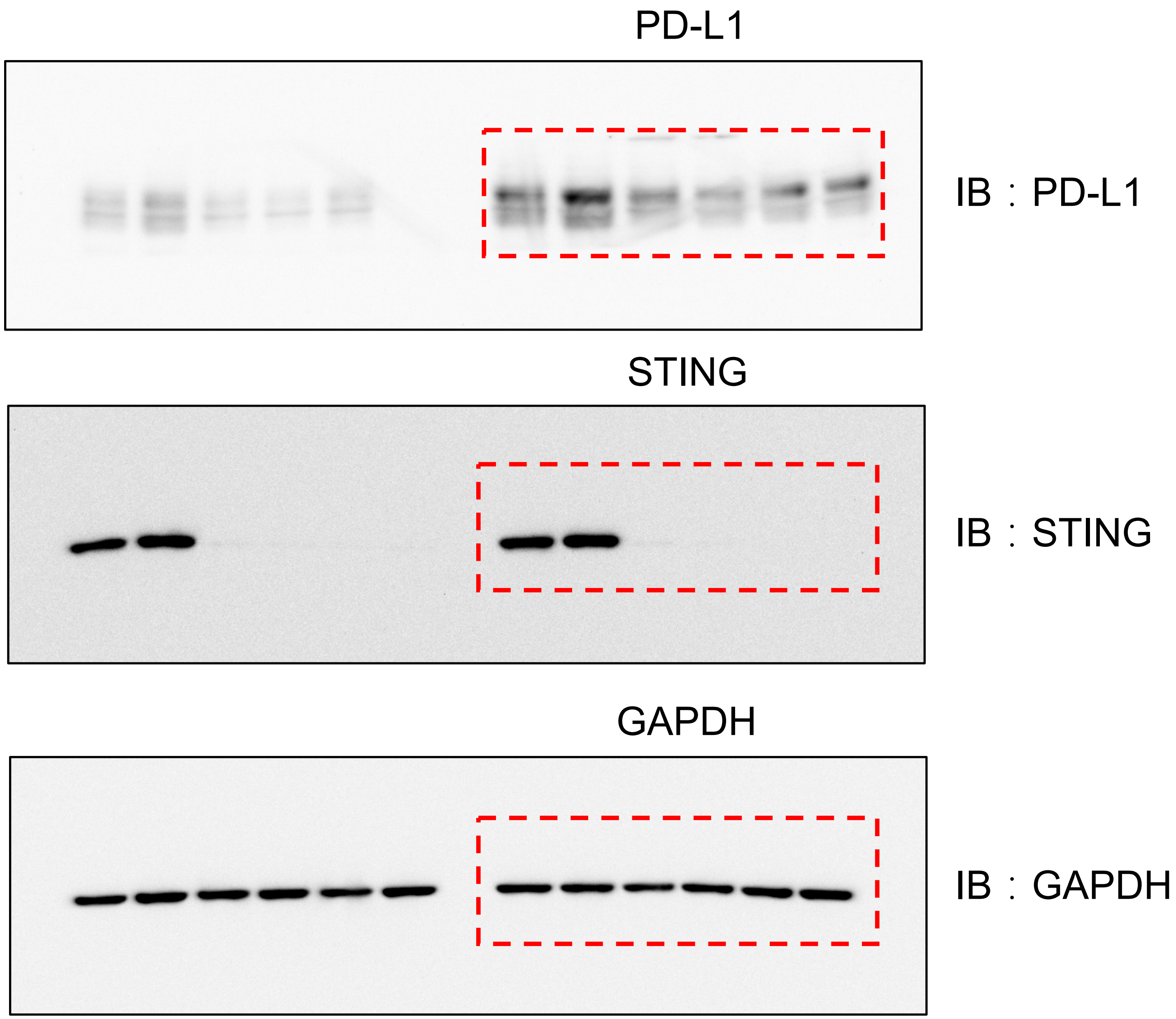

Full unedited gel for Figure 5A

PD-L1 and cGAS were detected separately in two gels by using the same biological samples. GAPDH in each gel was served as the loading control.

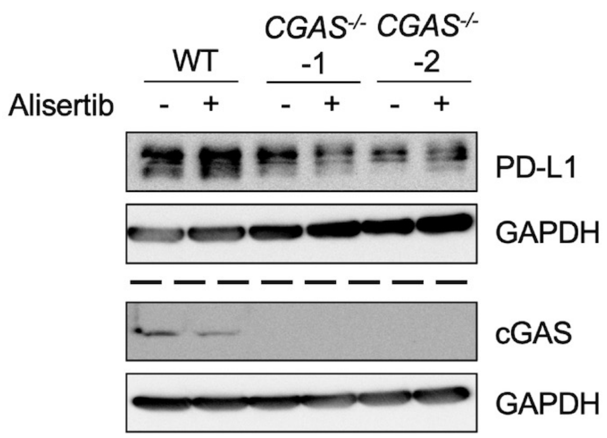

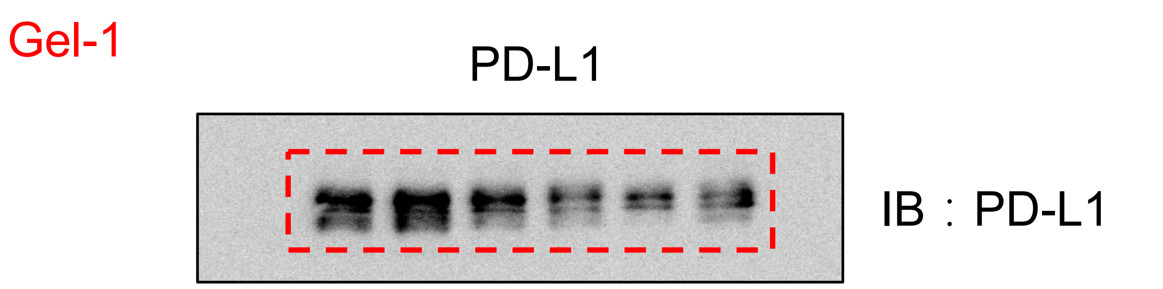

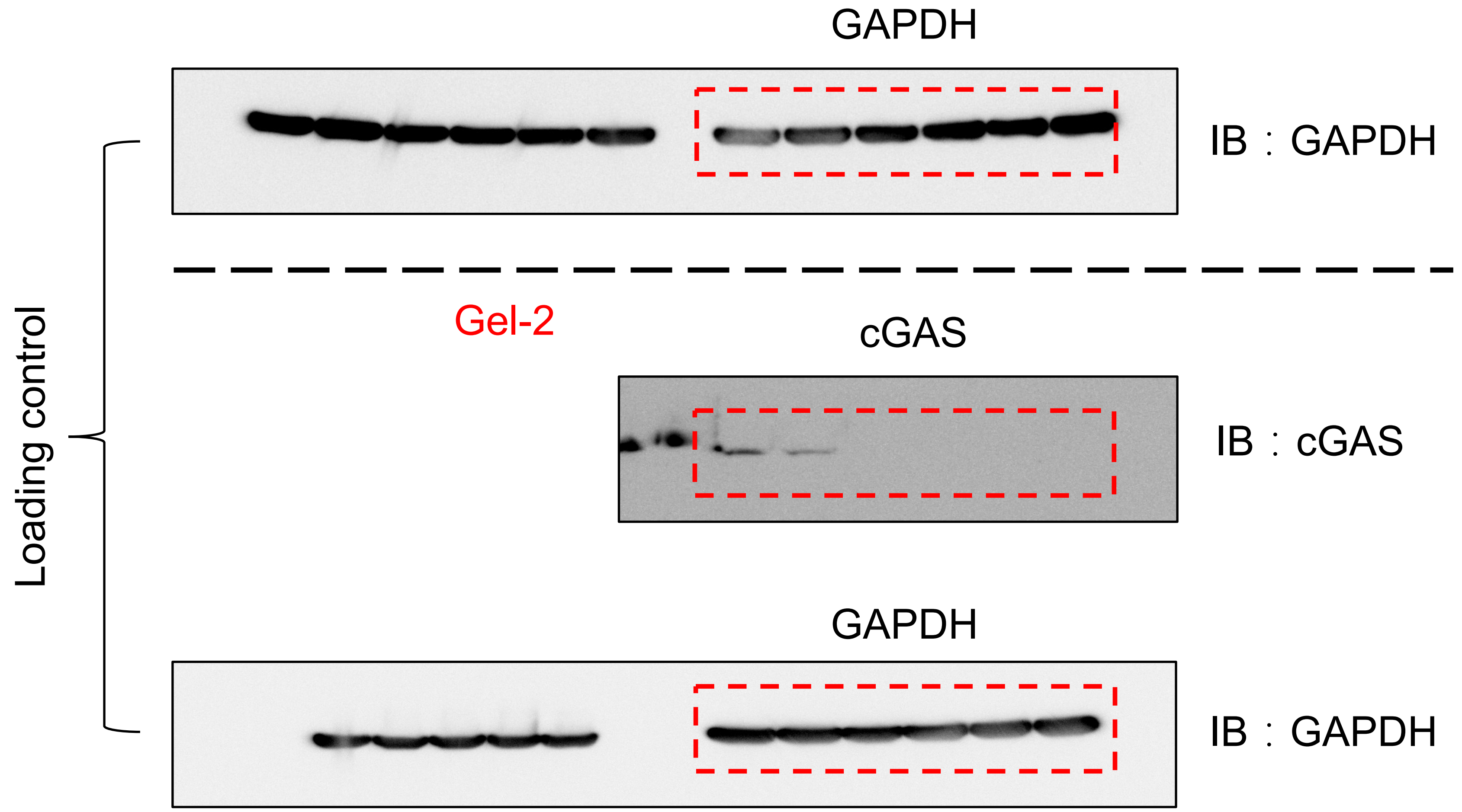

Full unedited gel for Figure 5D

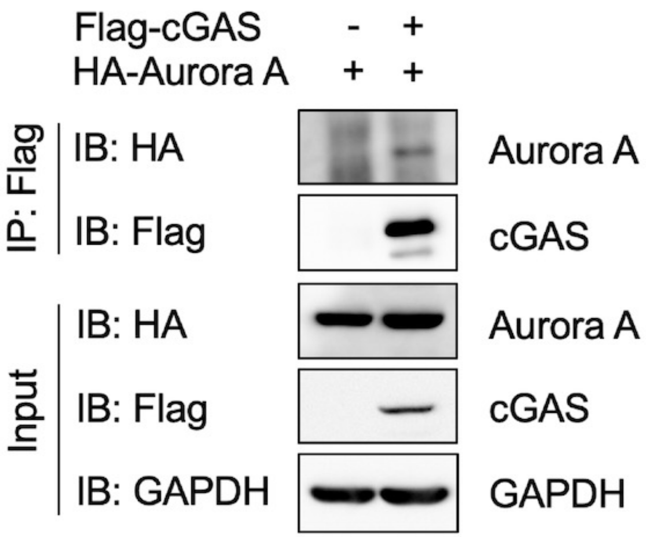

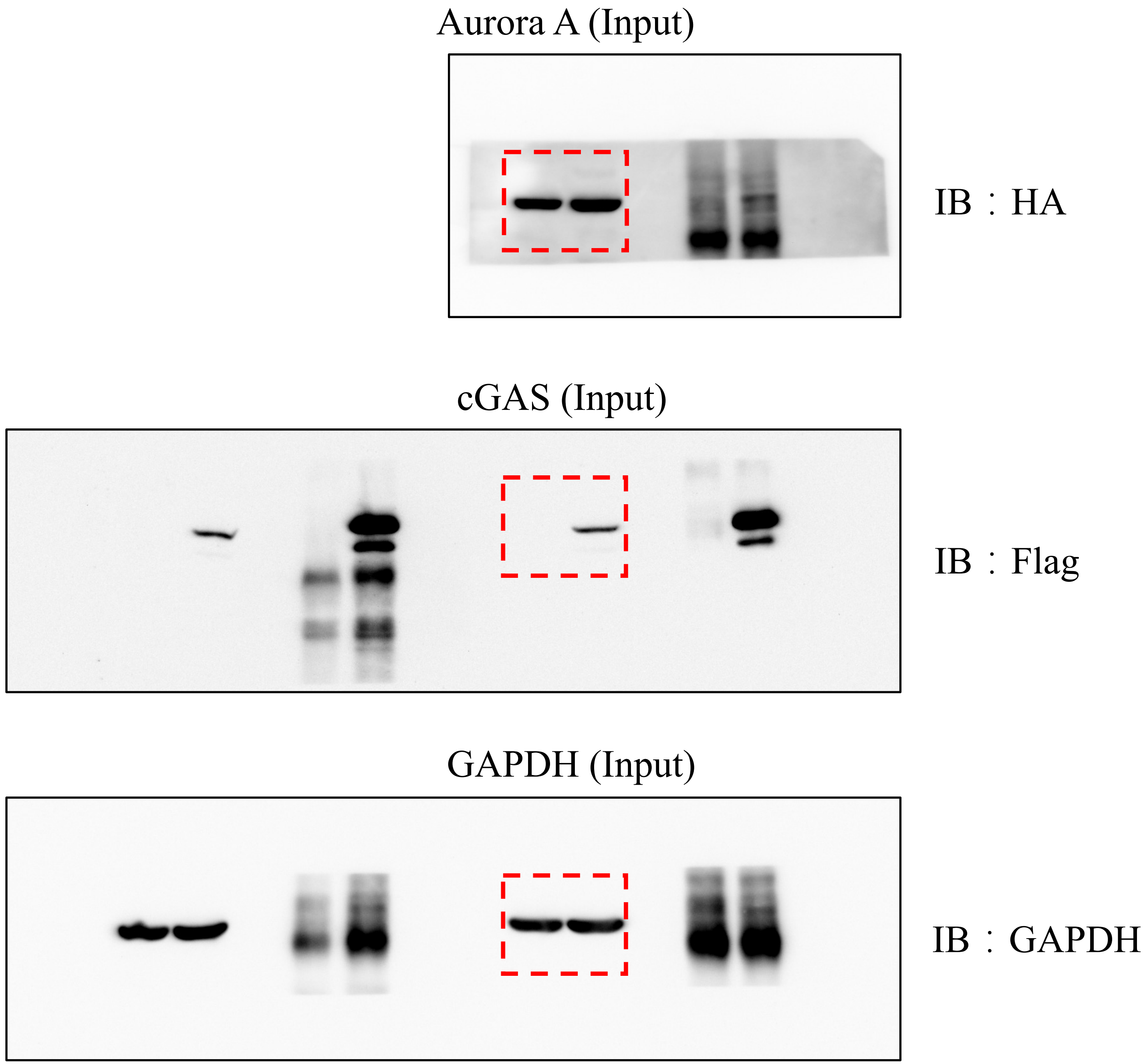

Full unedited gel for Figure 5D

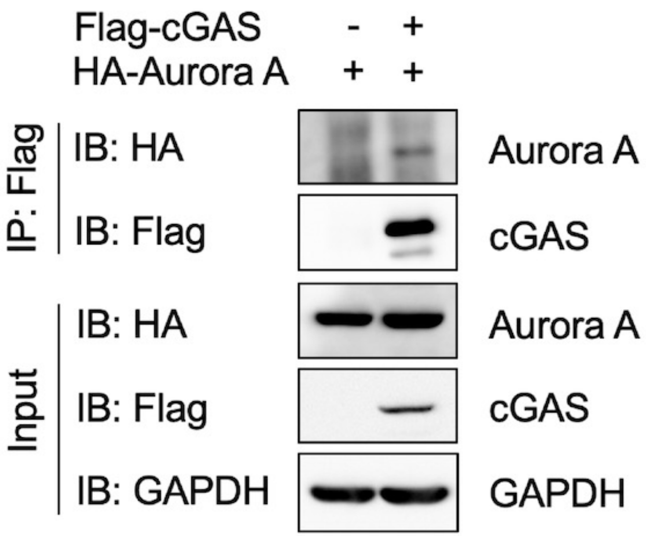

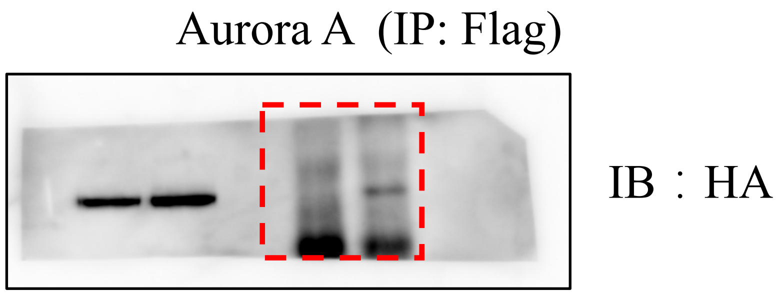

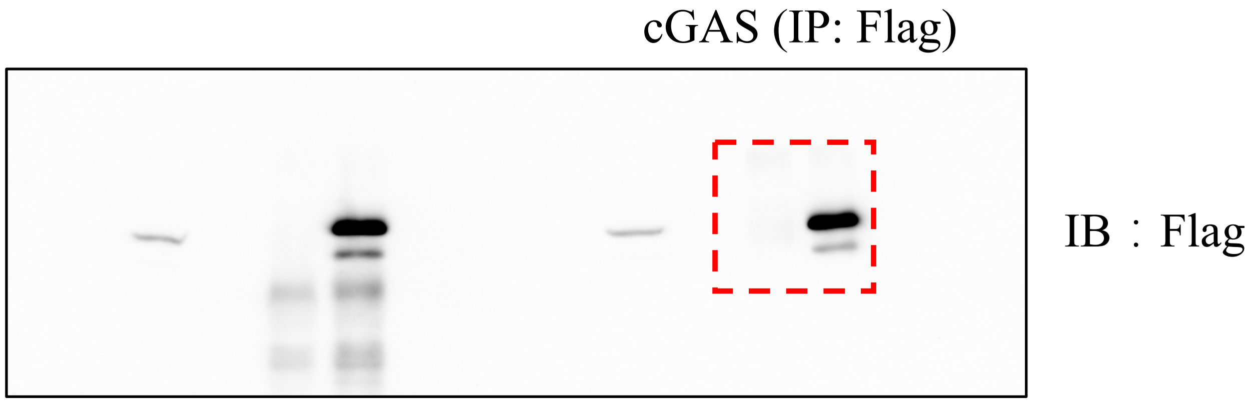

Full unedited gel for Figure 5E

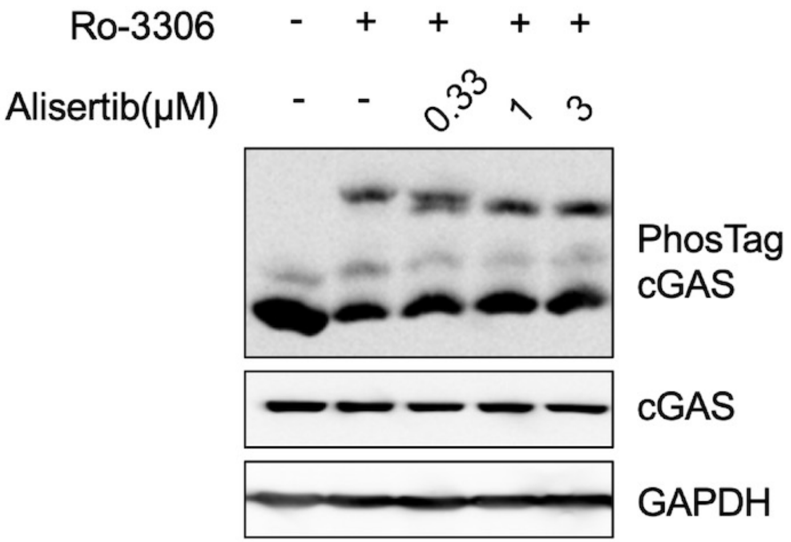

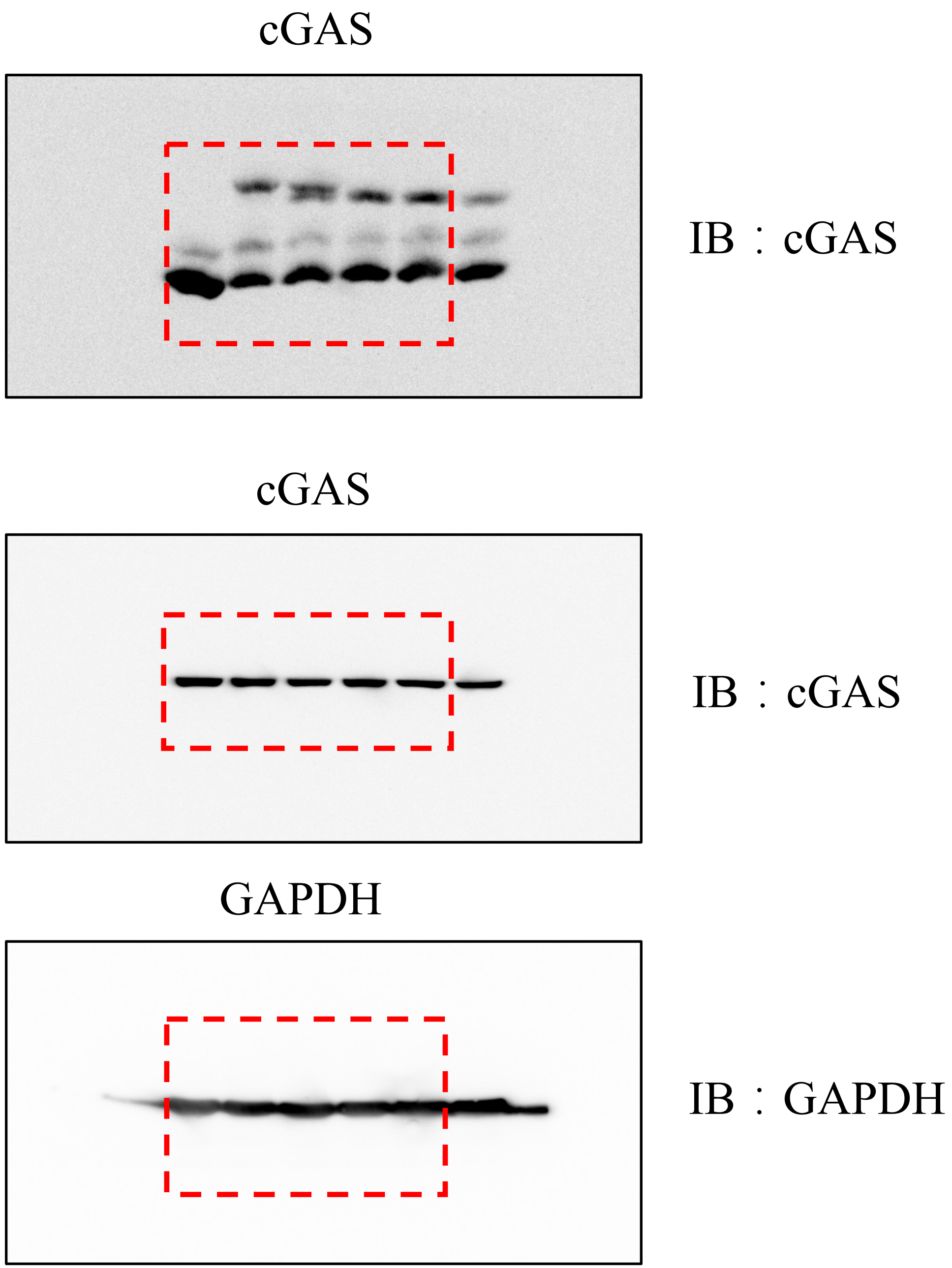

Full unedited gel for Figure S5A

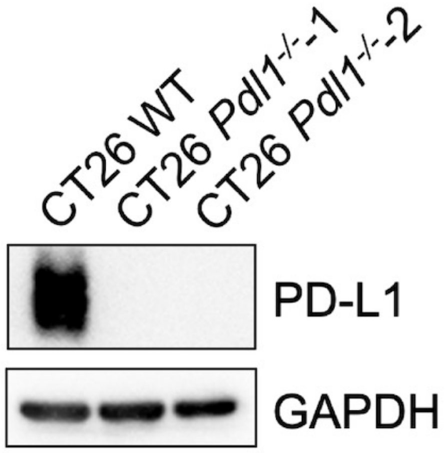

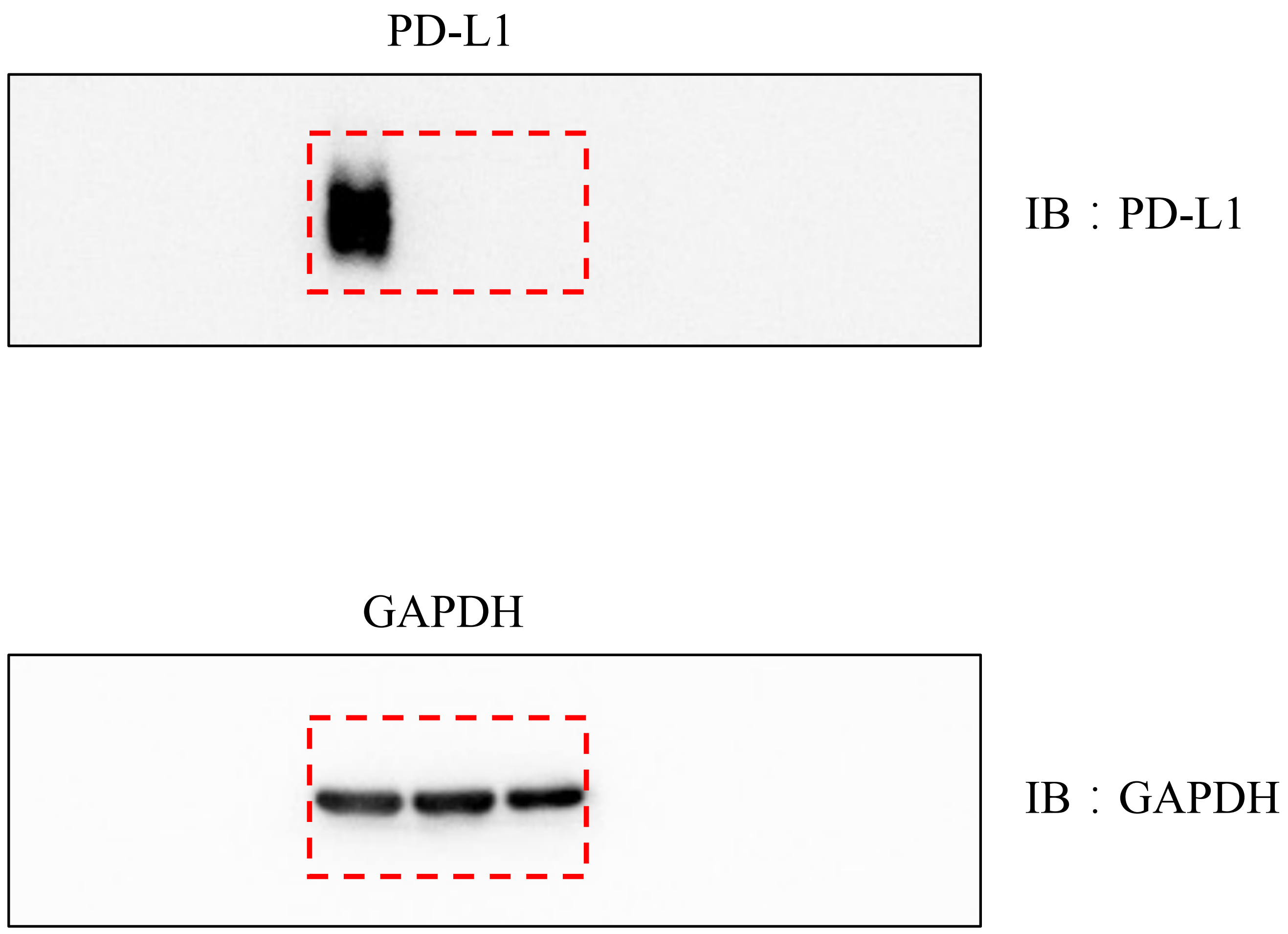

Full unedited gel for Figure S6A

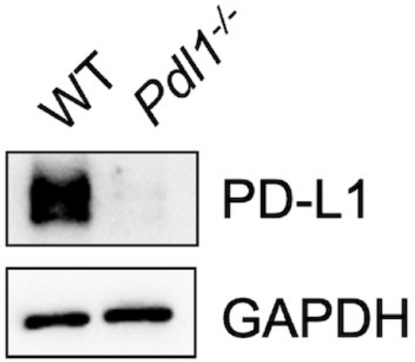

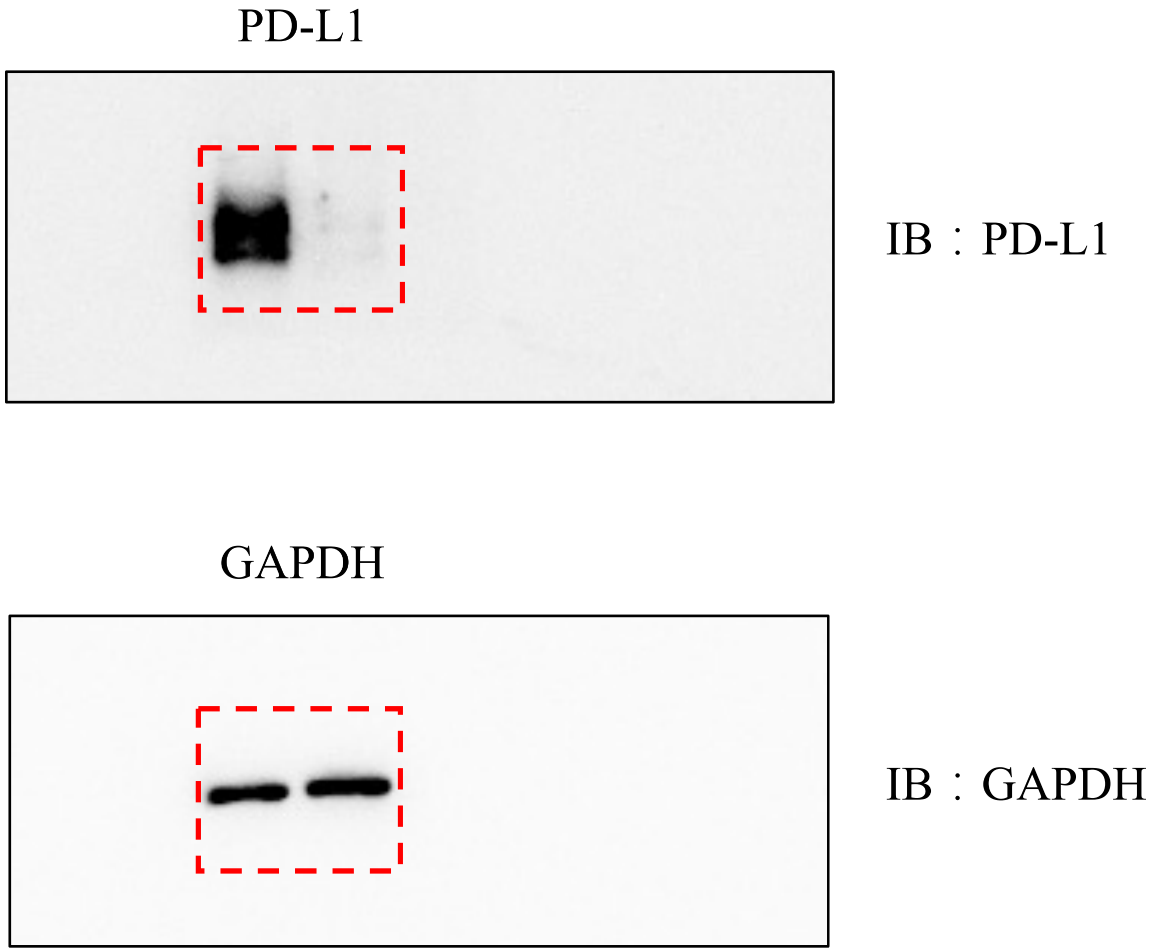

Full unedited gel for Figure S9A

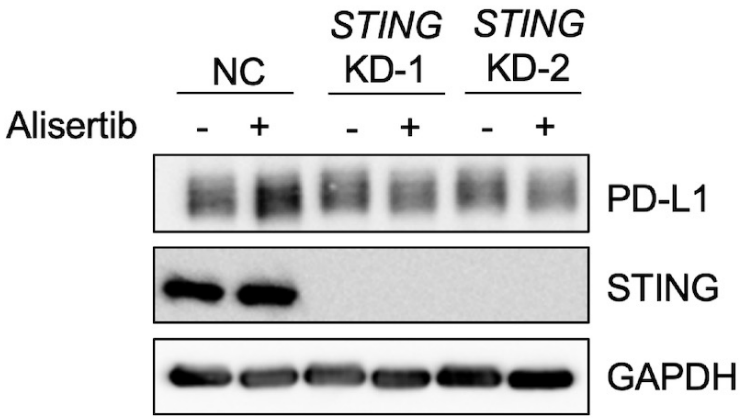

PD-L1

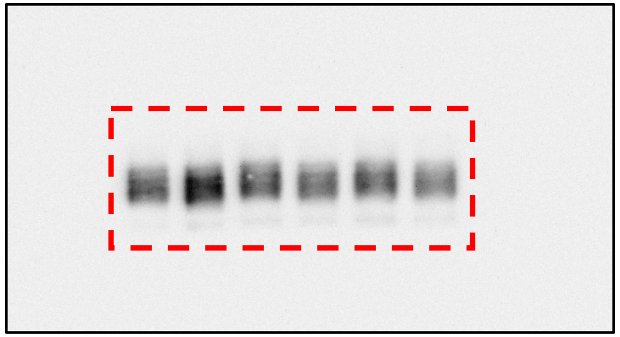

IB：PD-L1

STING

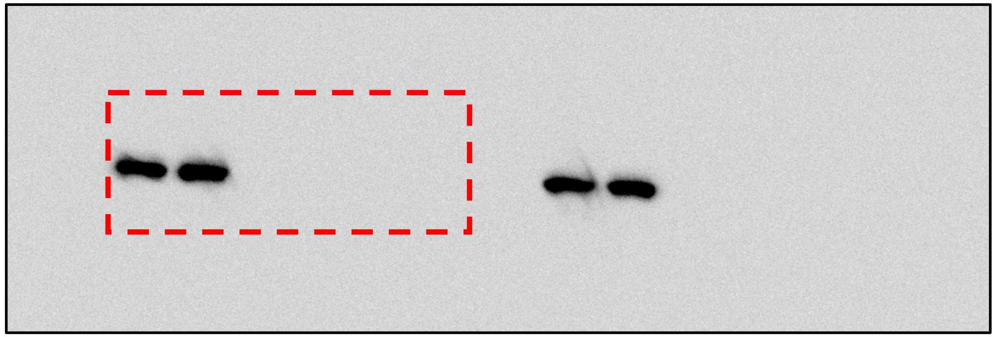

IB：STING

GAPDH

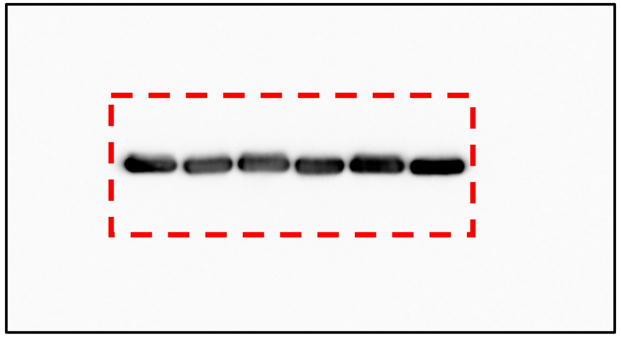

IB：GAPDH

Full unedited gel for Figure S11A

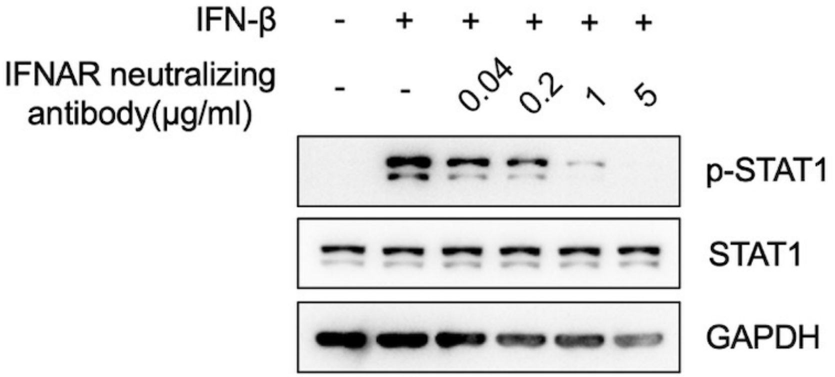

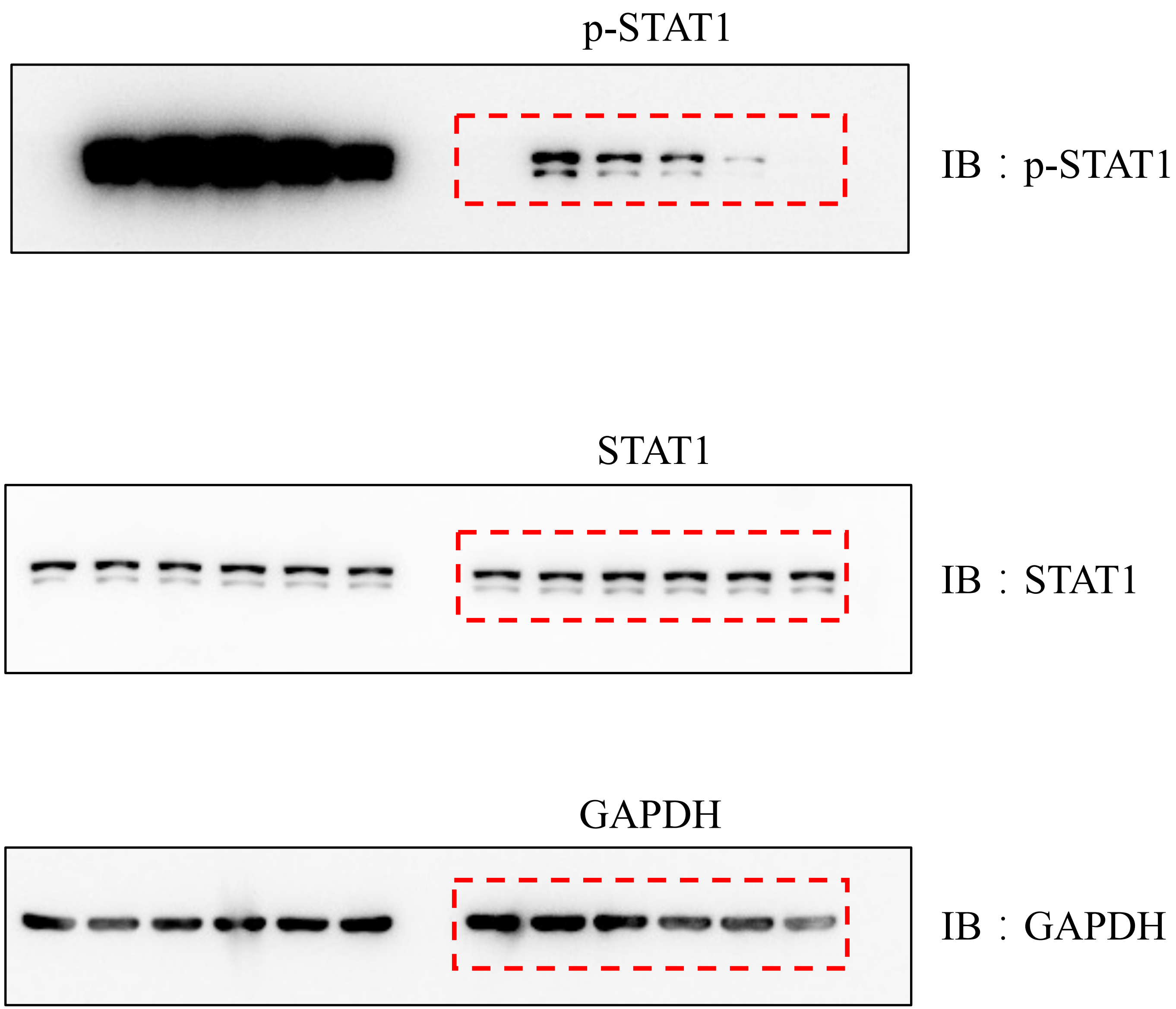

Full unedited gel for Figure S11C

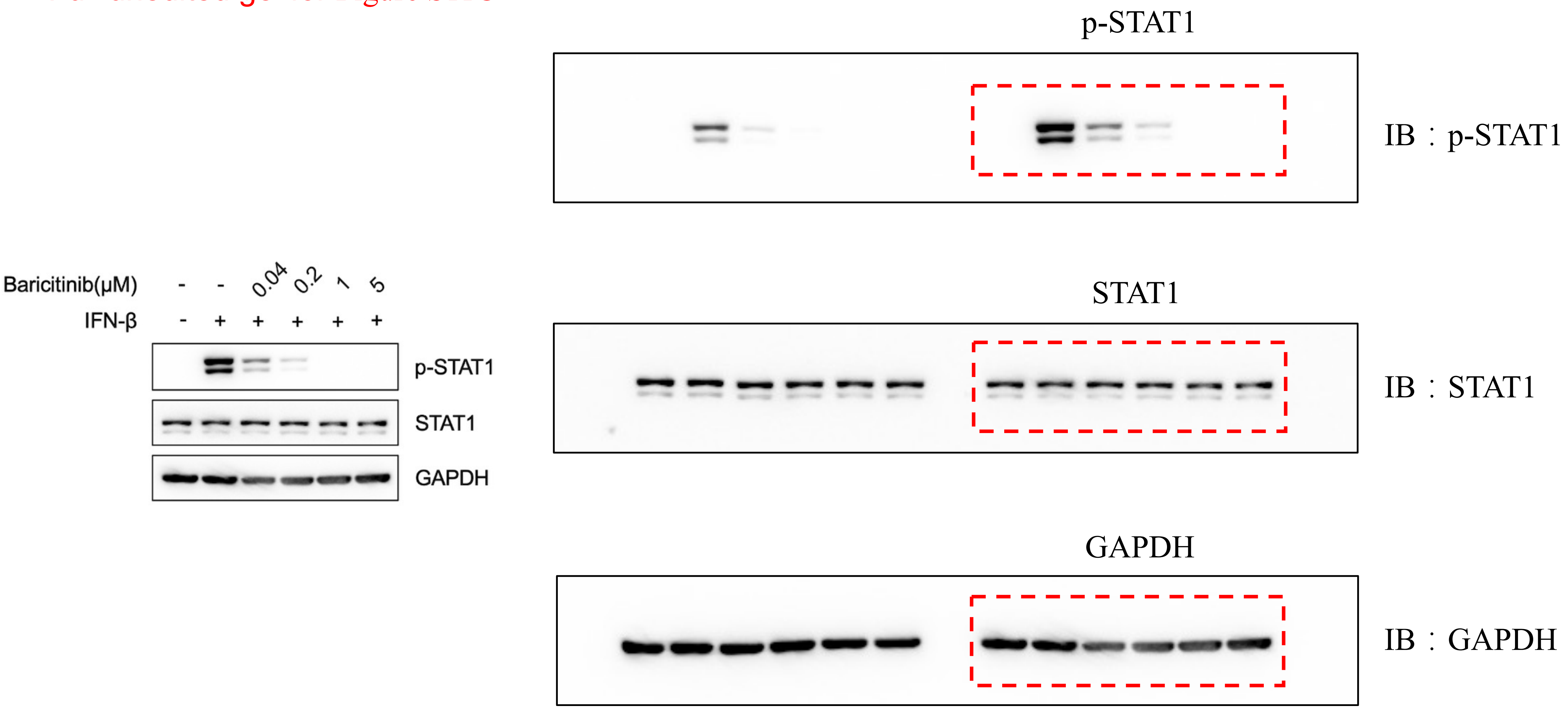

Full unedited gel for Figure S12A

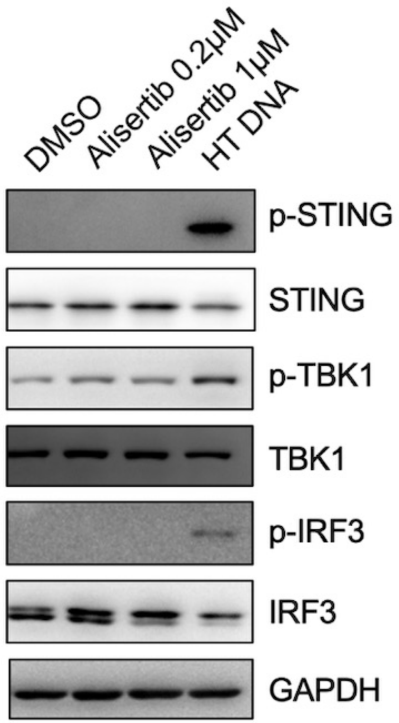

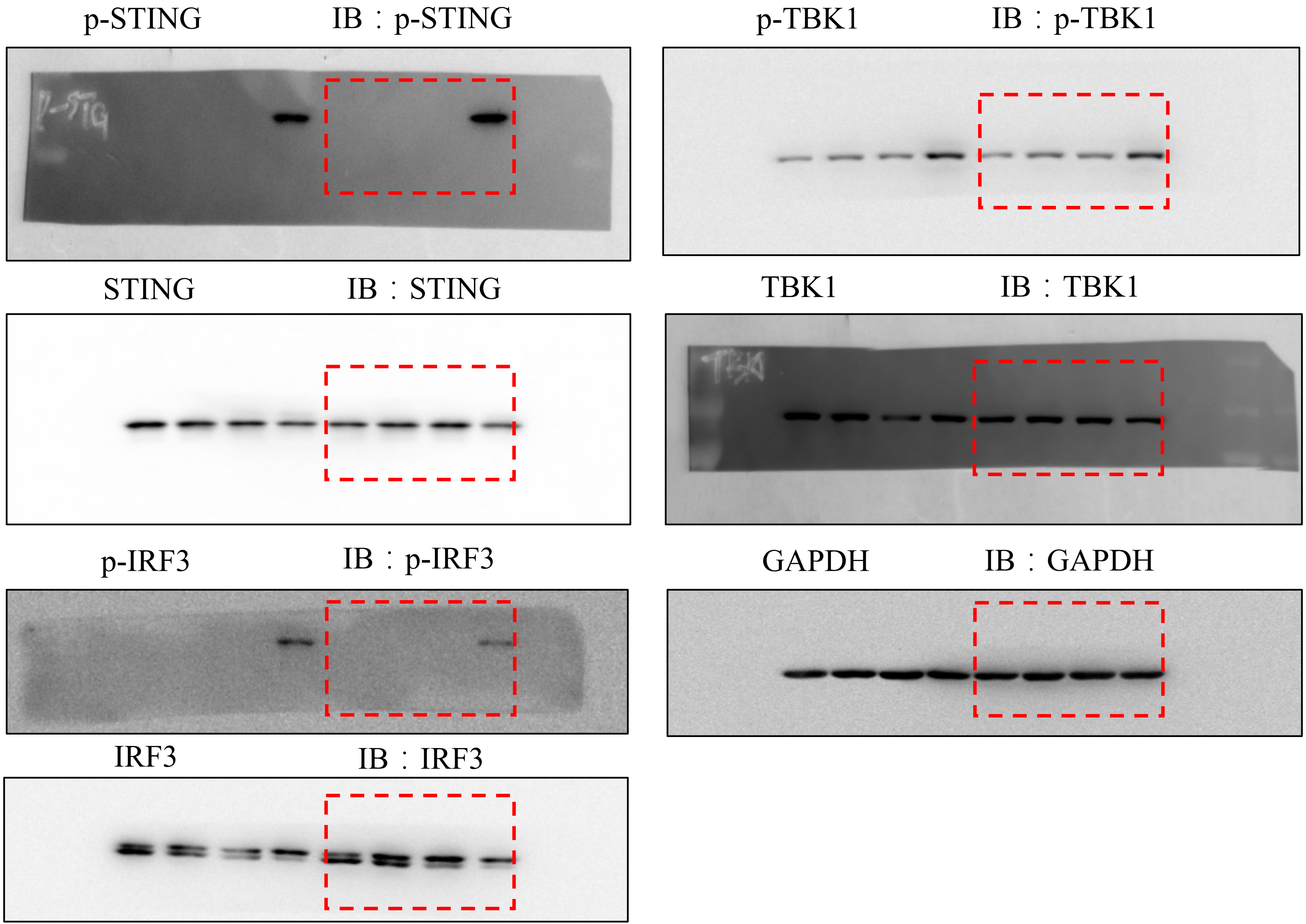

Full unedited gel for Figure S13A

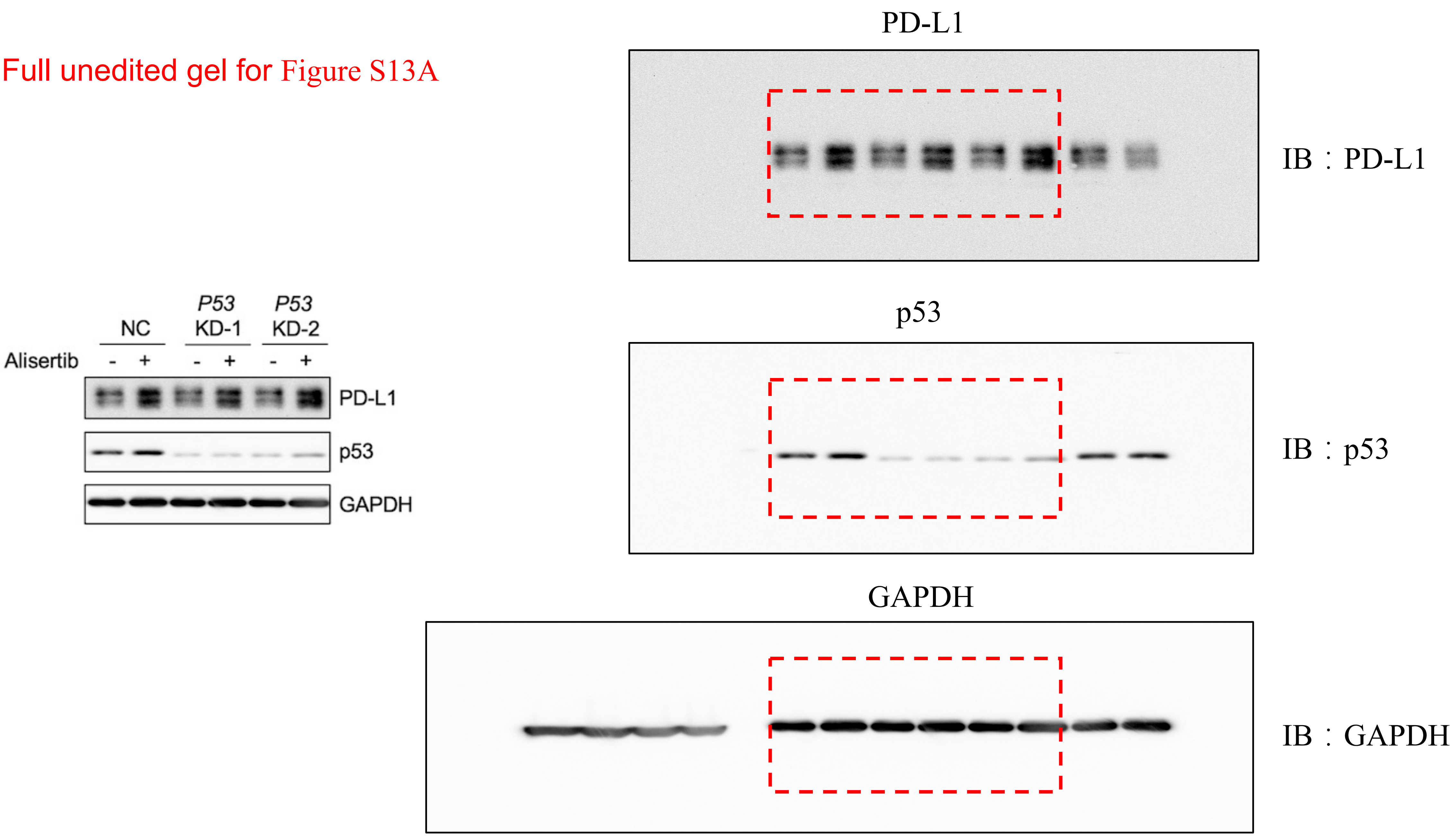

Full unedited gel for Figure S13E

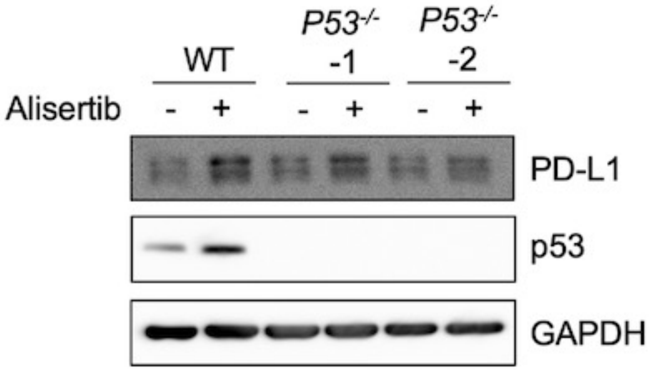

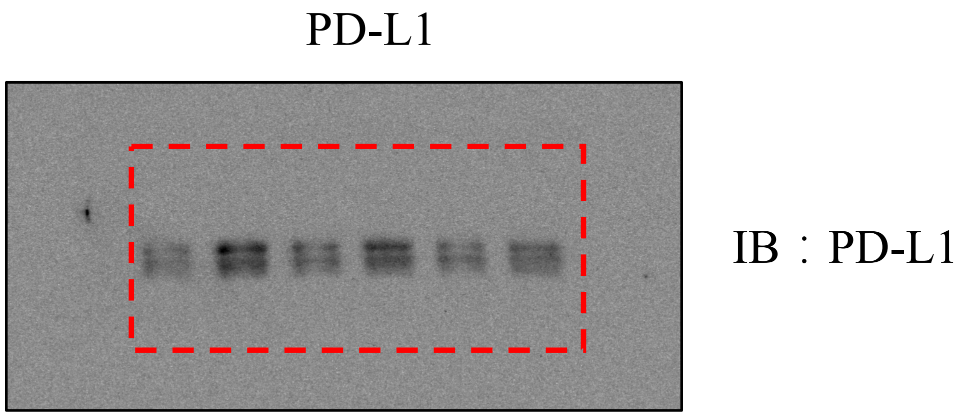

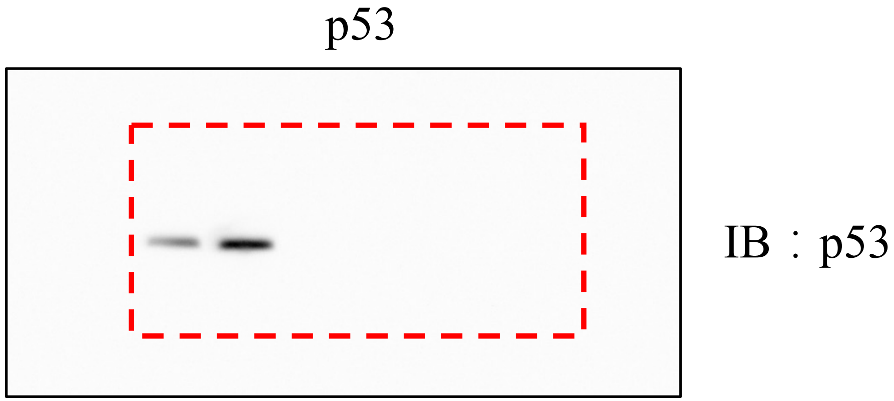

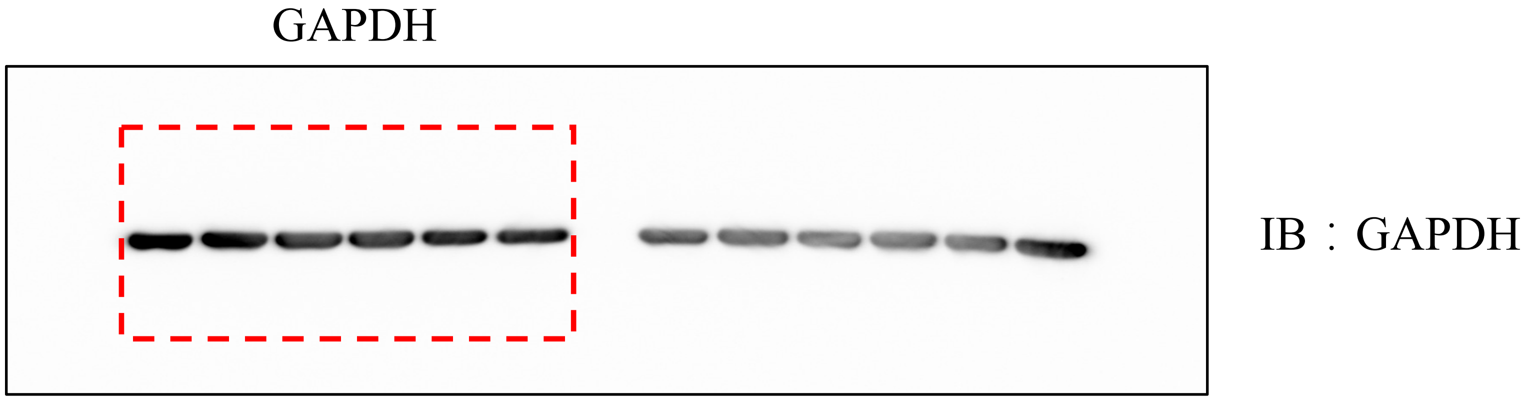

Full unedited gel for Figure S14A

PD-L1 and cGAS were detected separately in two gels by using the same biological samples. GAPDH in each gel was served as the loading control.

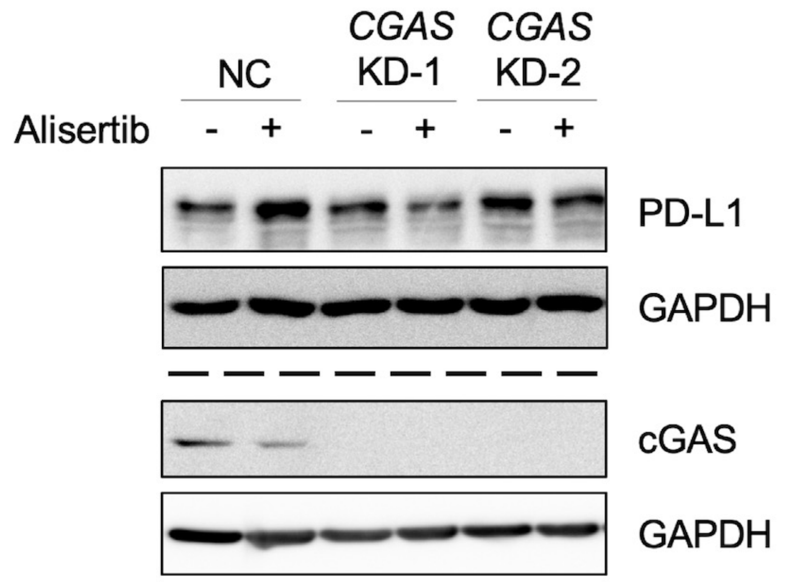

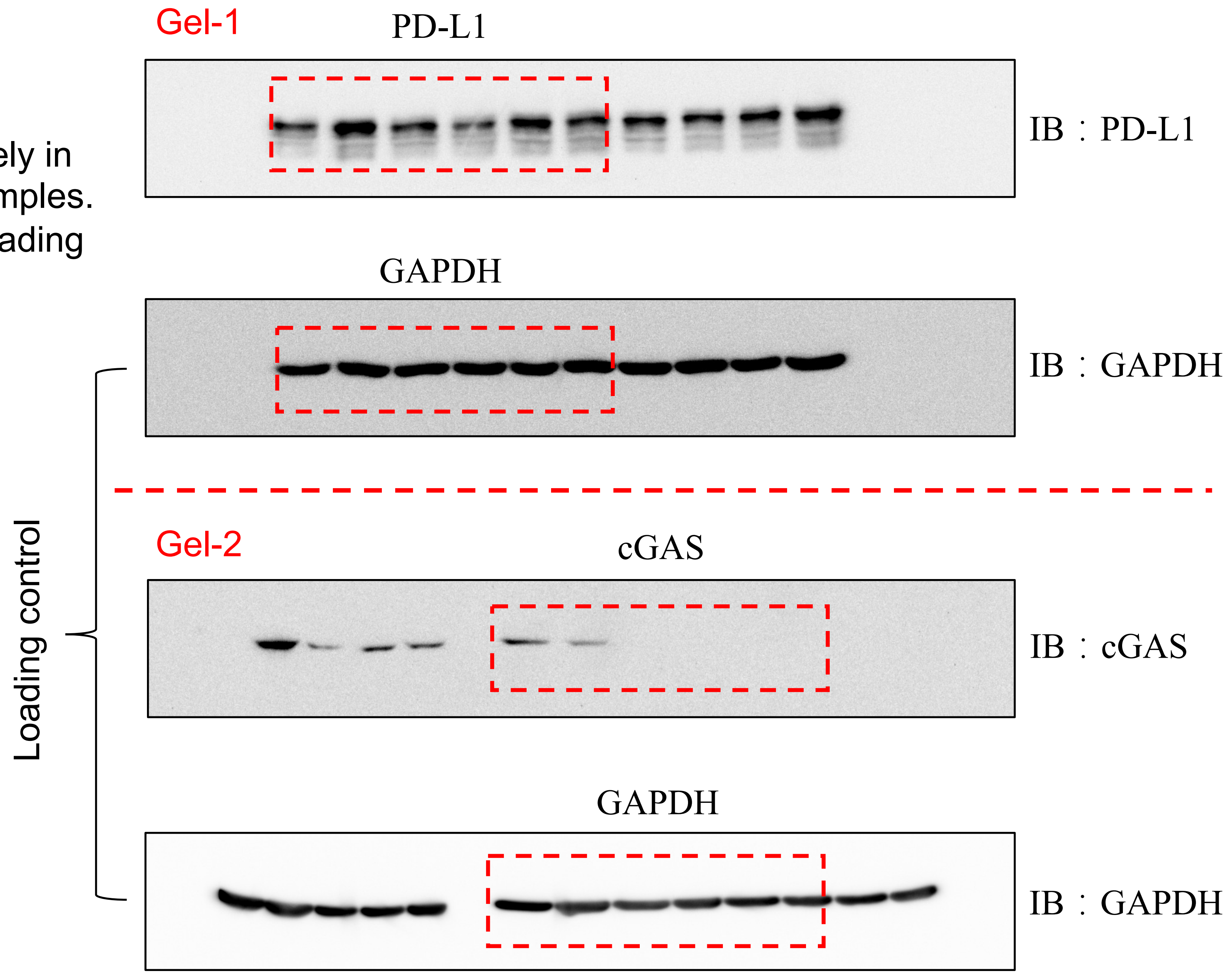

Full unedited gel for Figure S17A

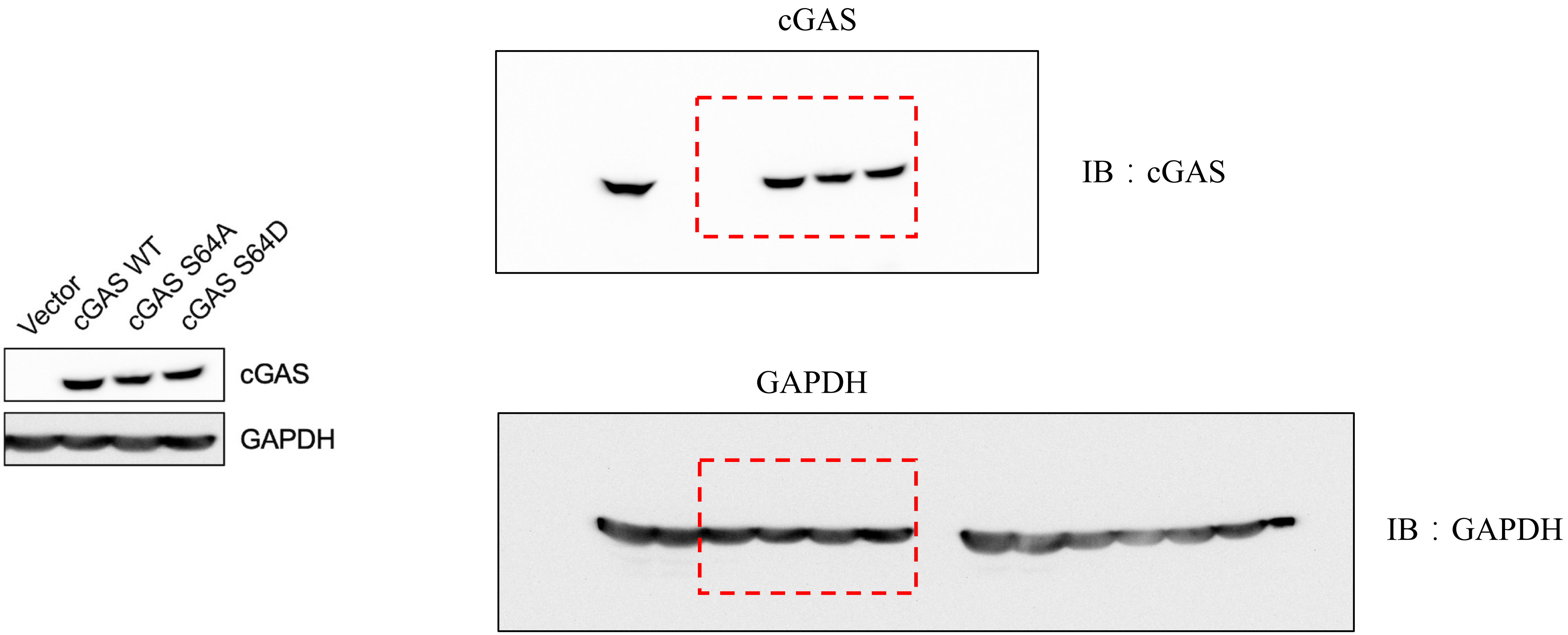

Supplement: Supplemental data [file jci-133-161929-s066.pdf]
